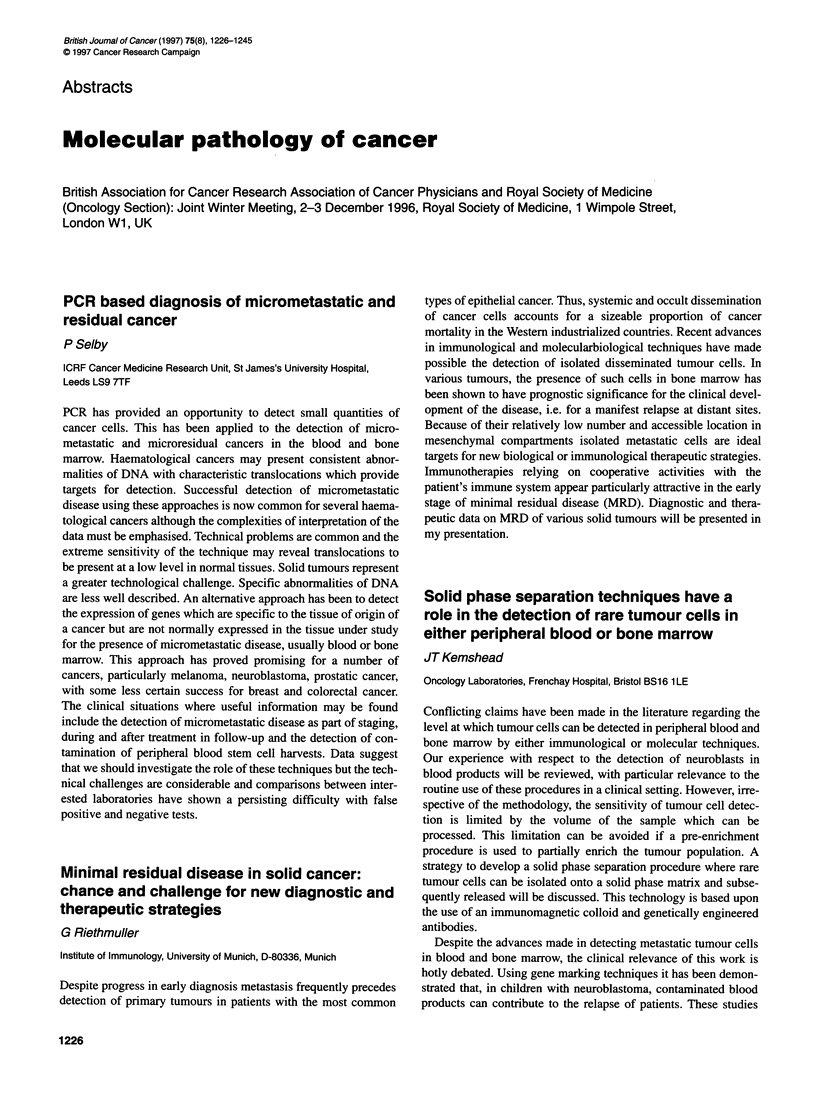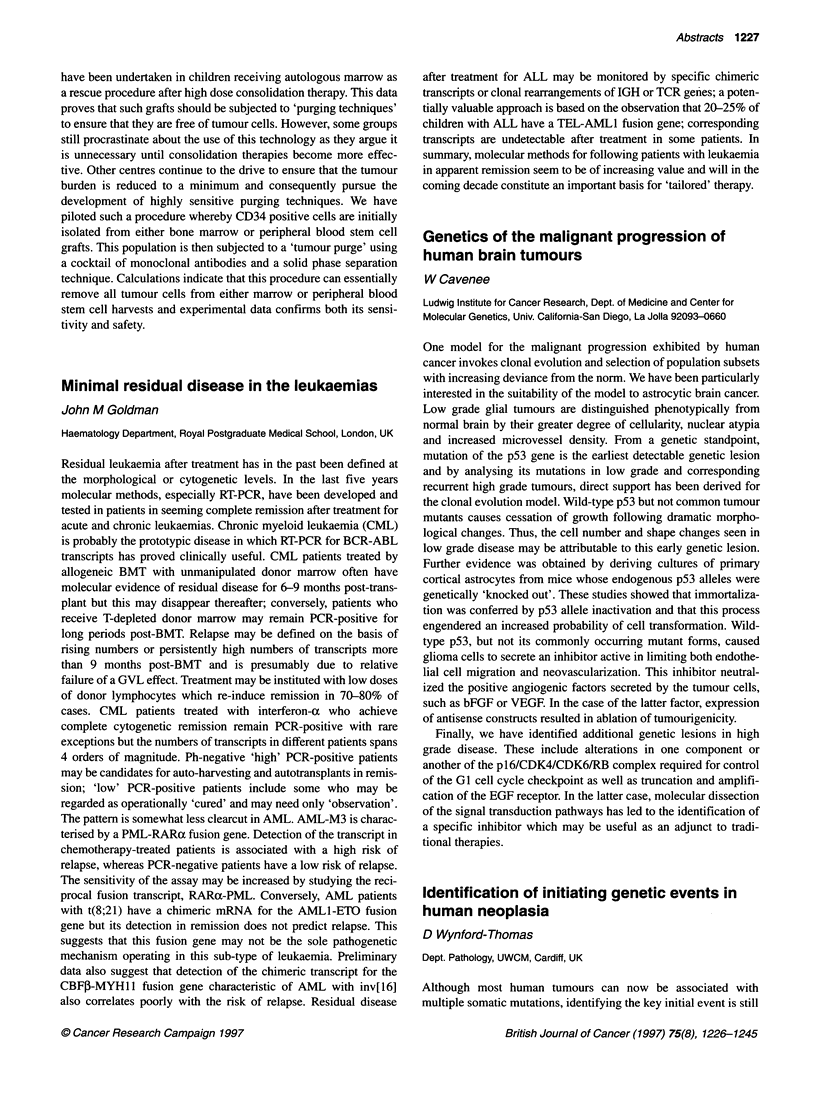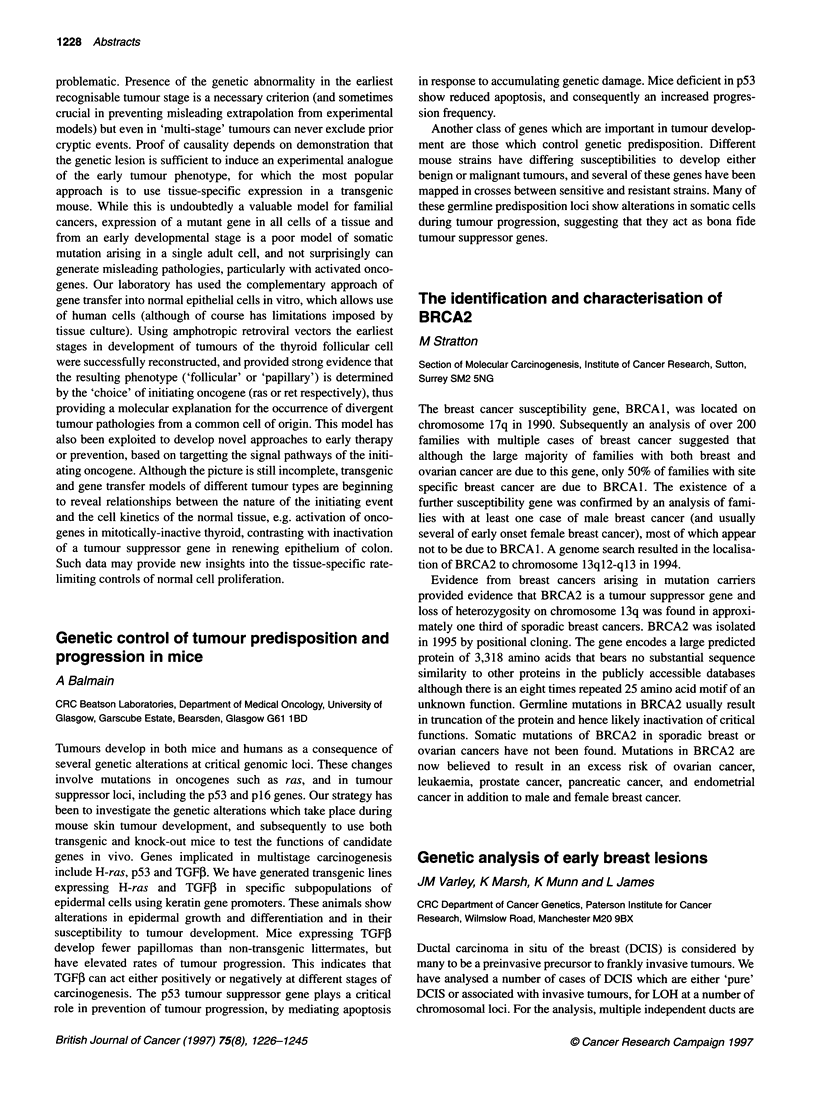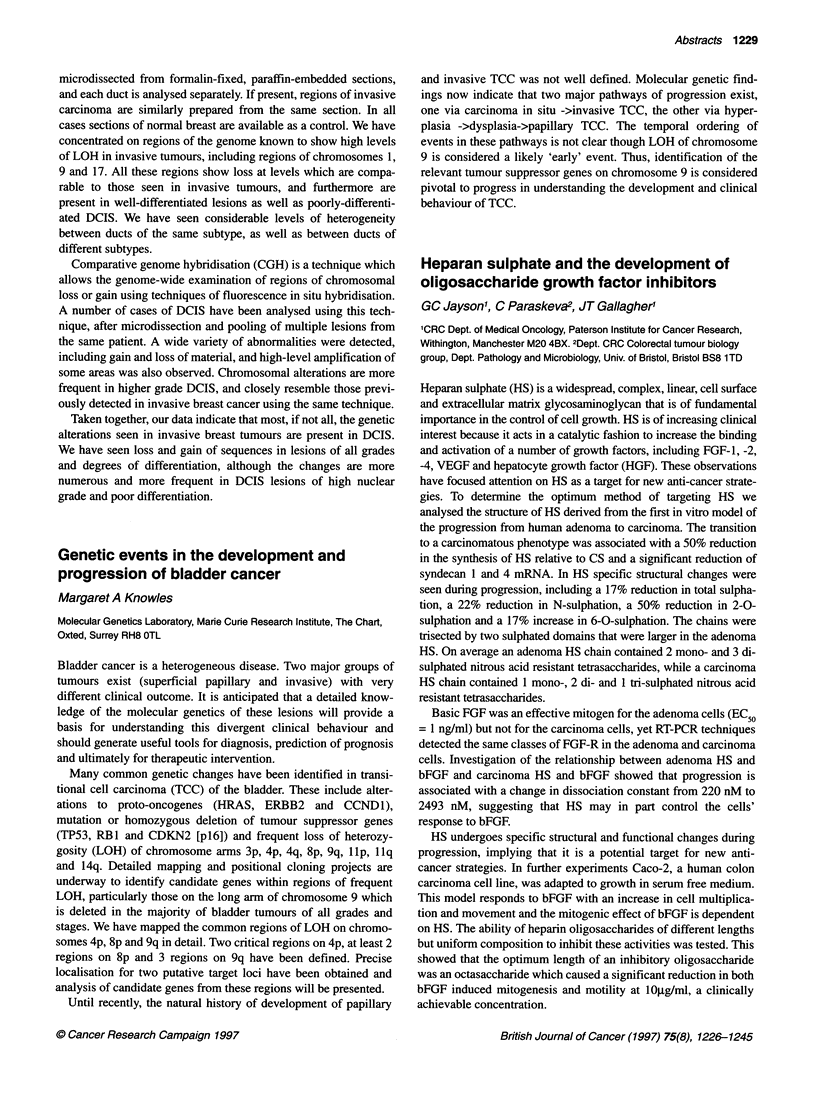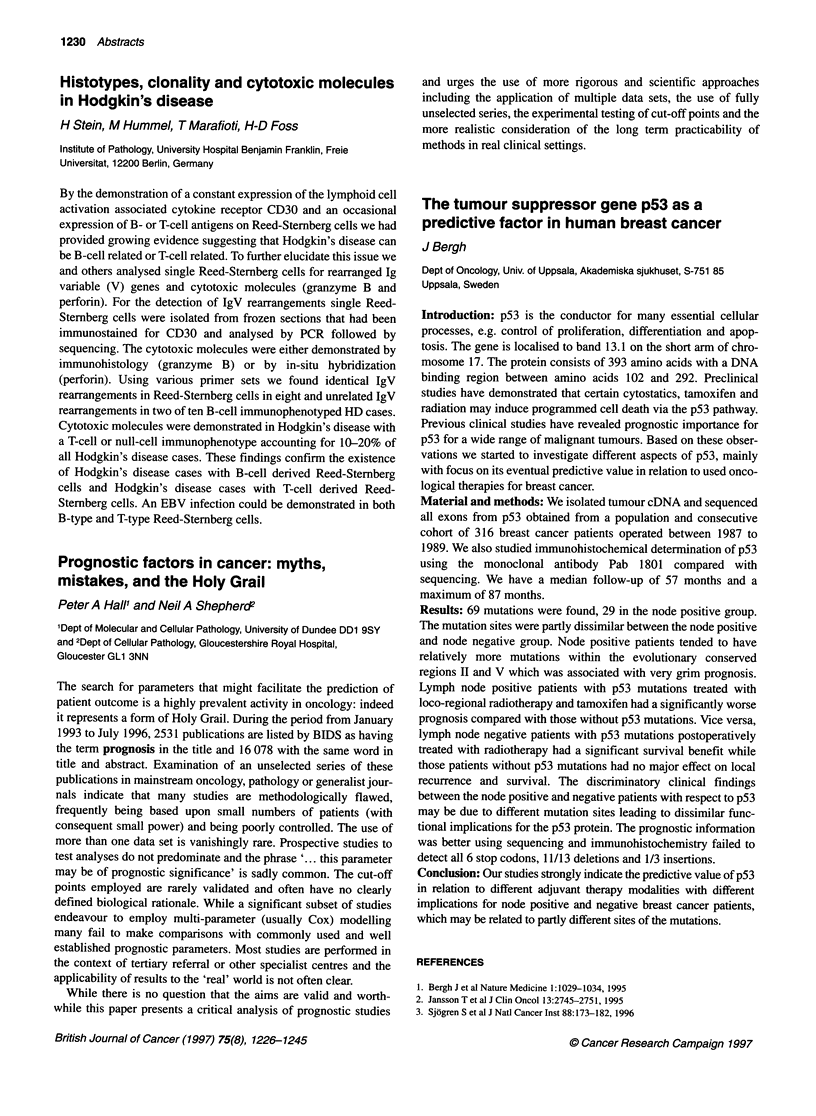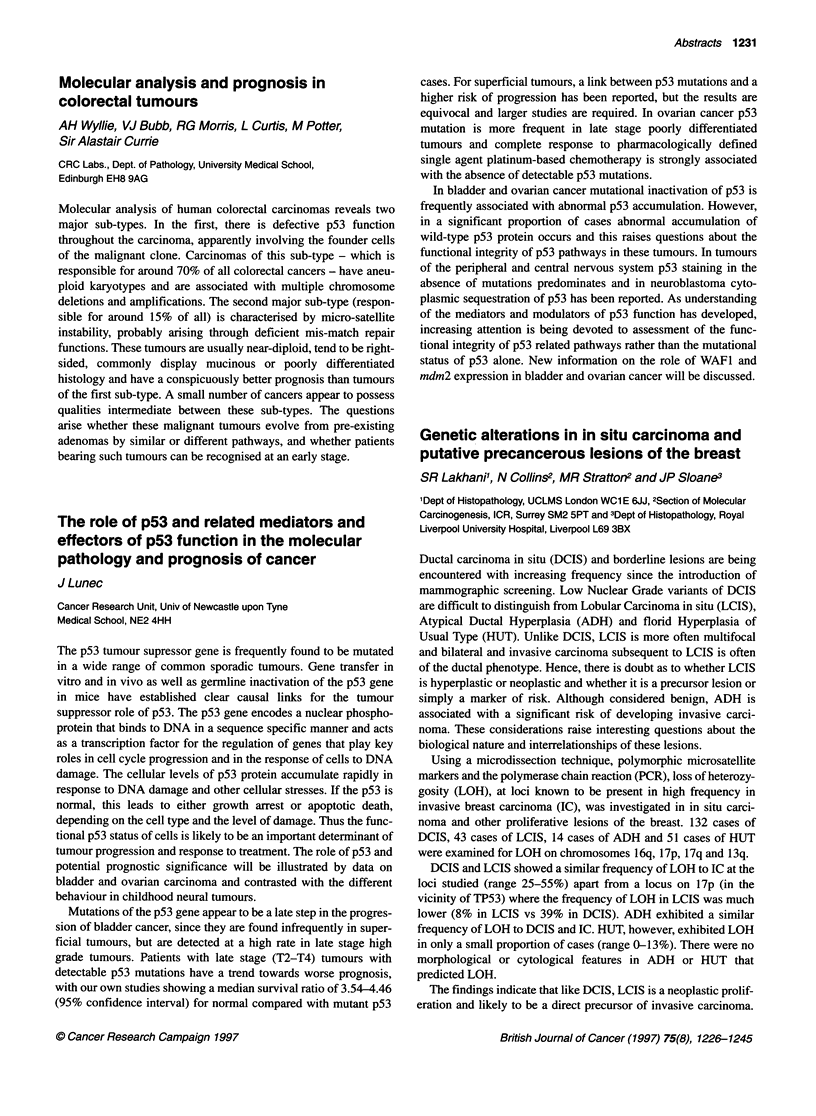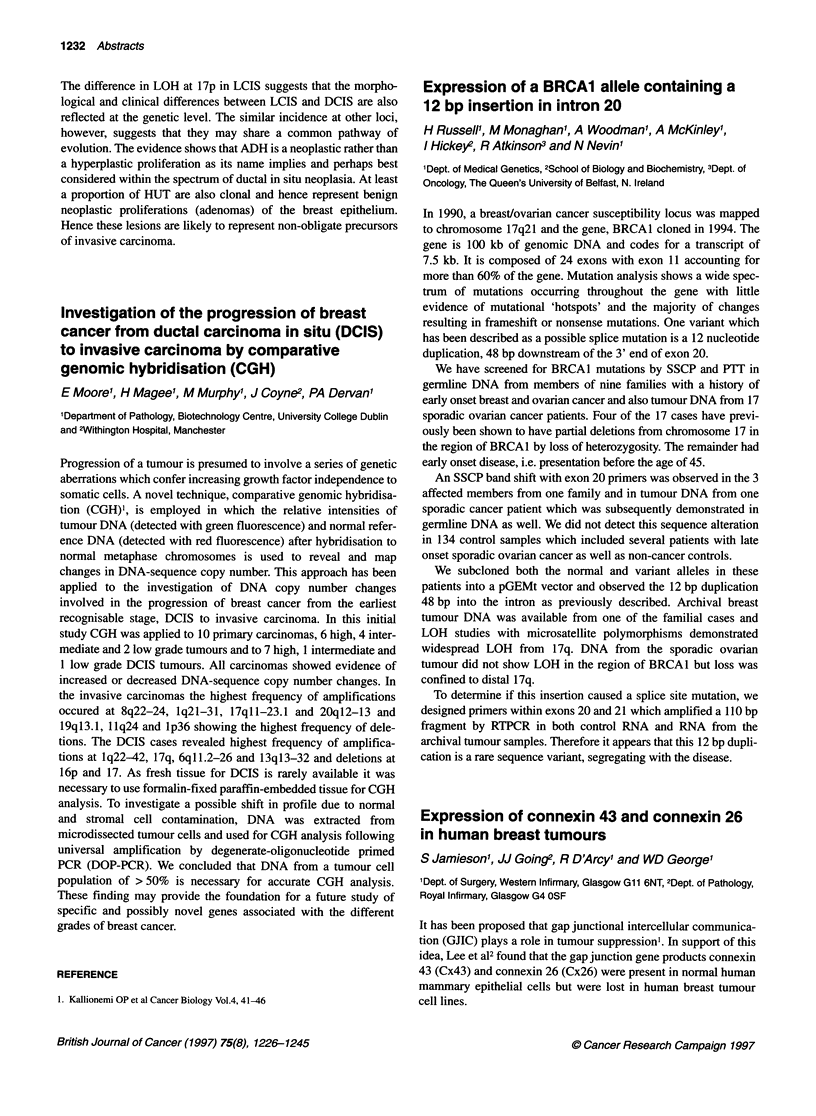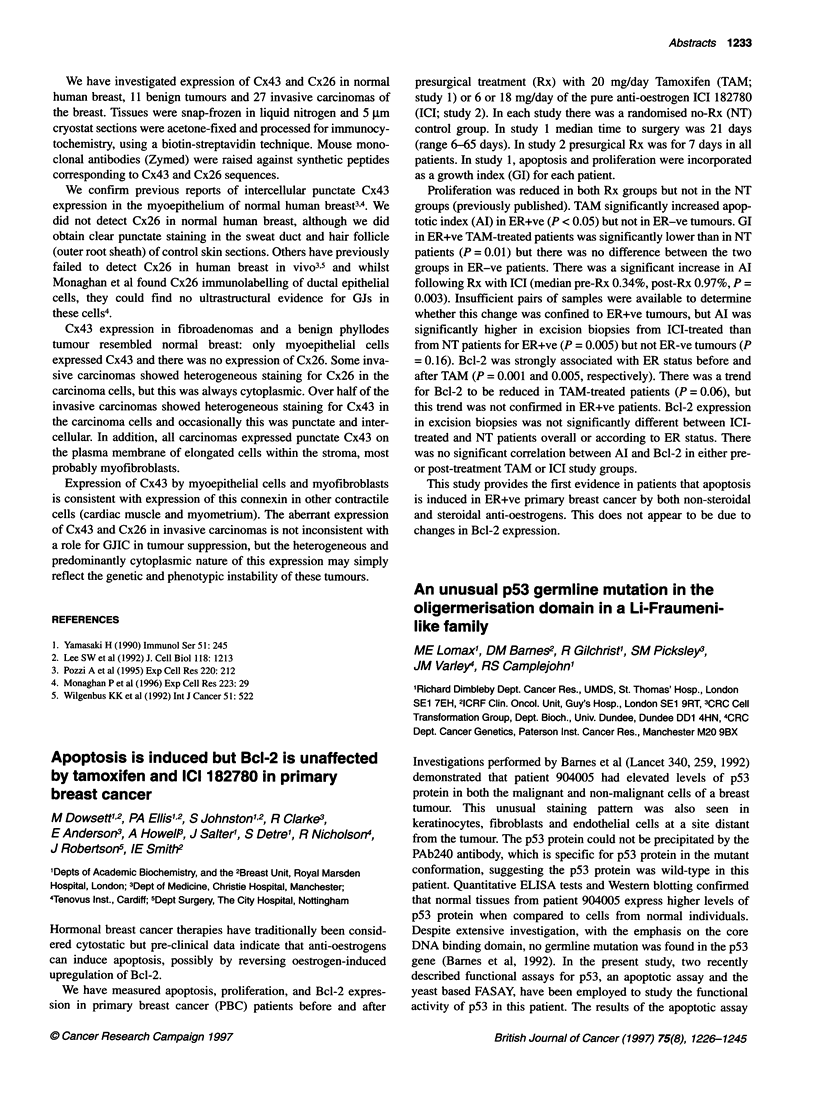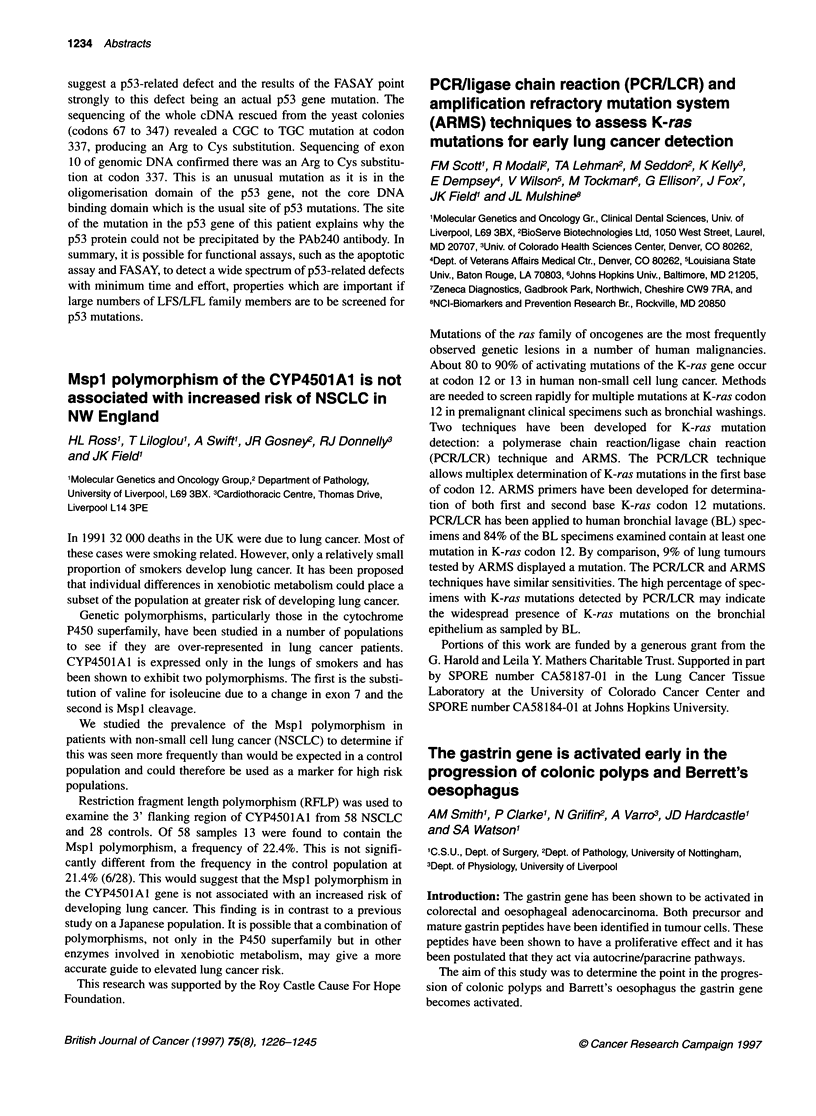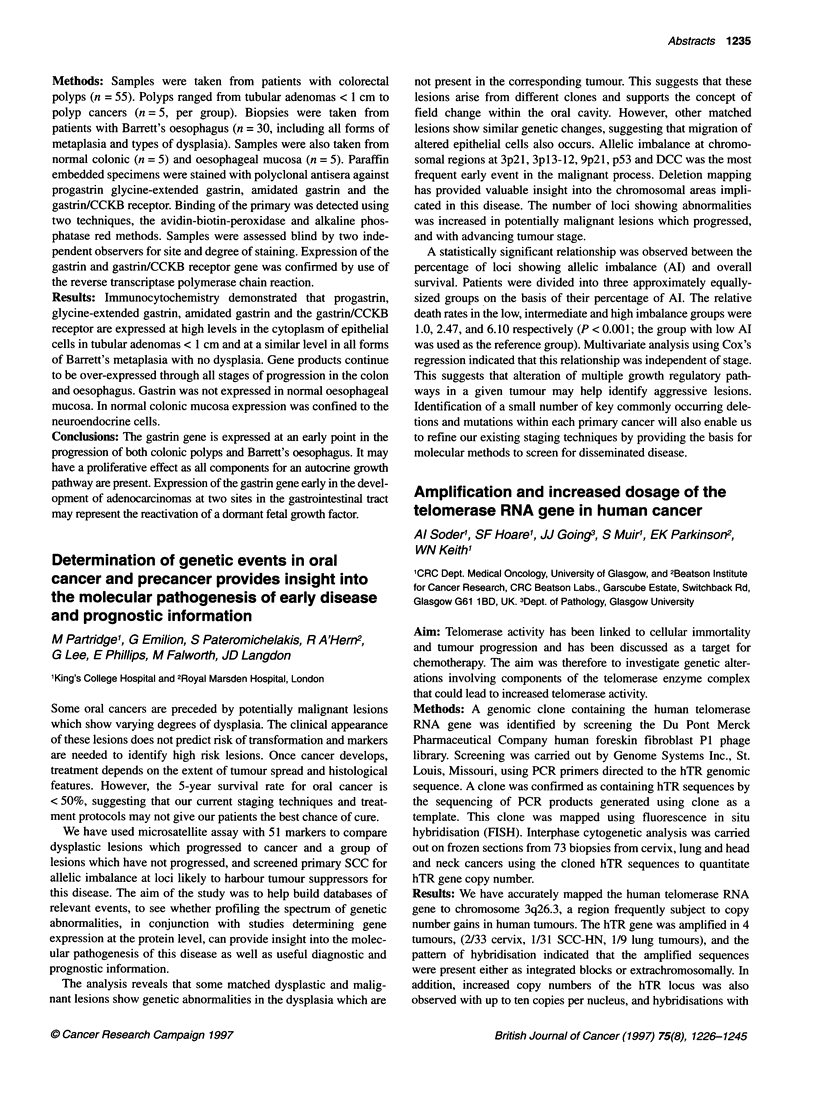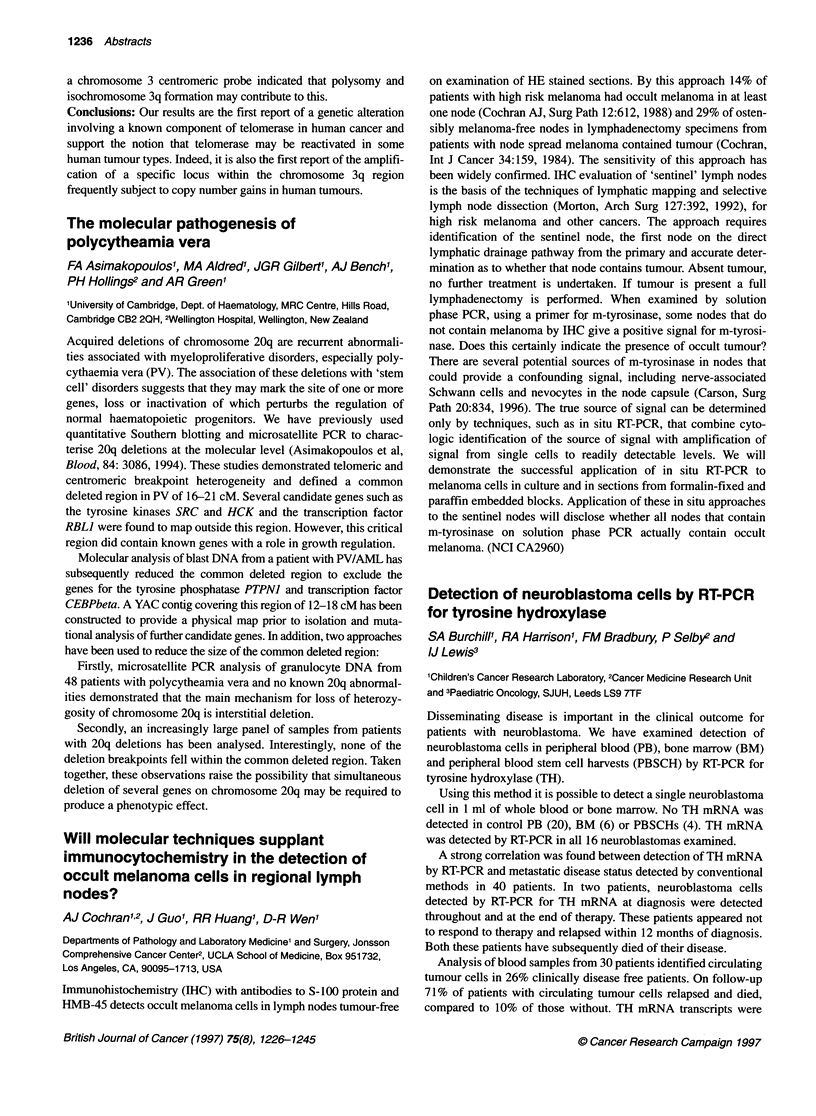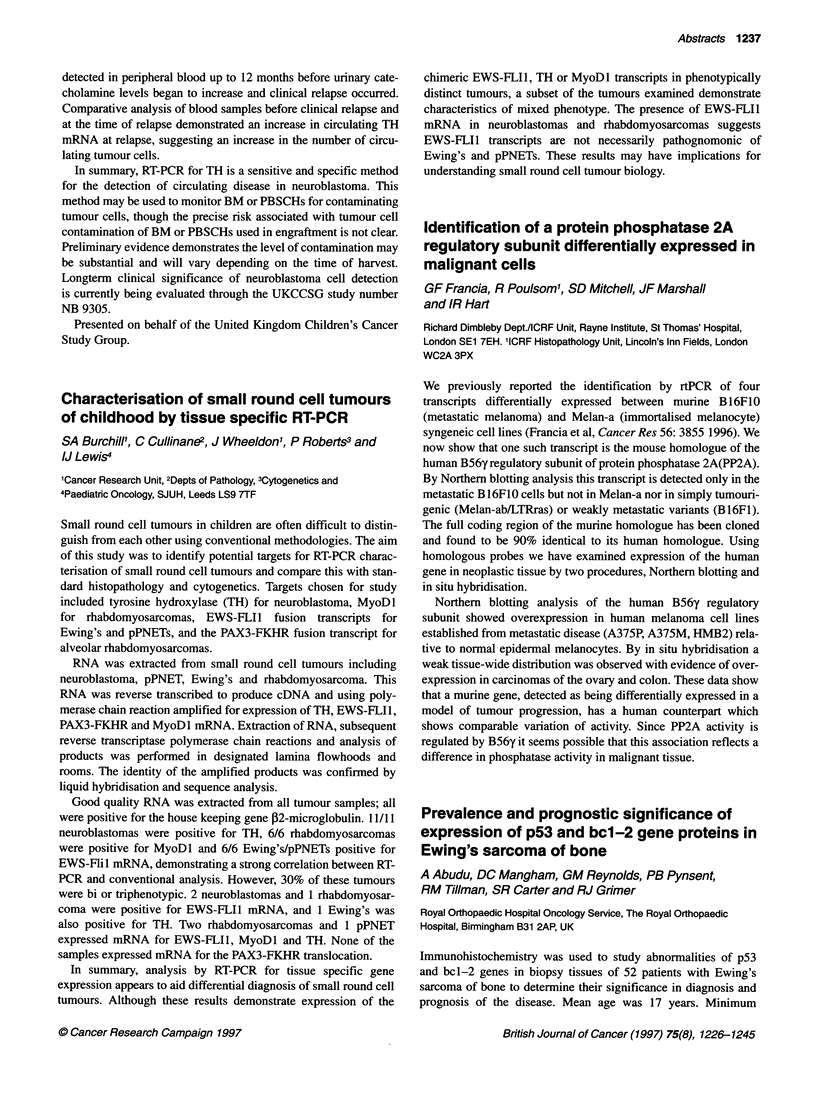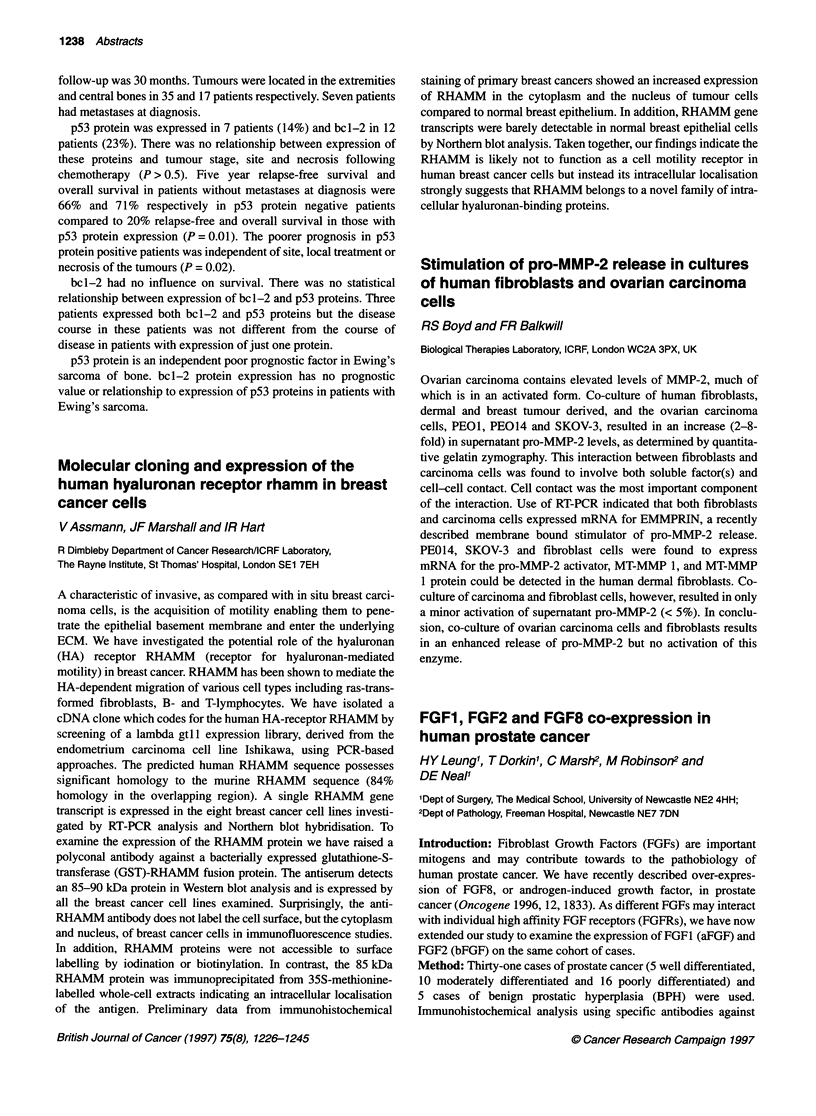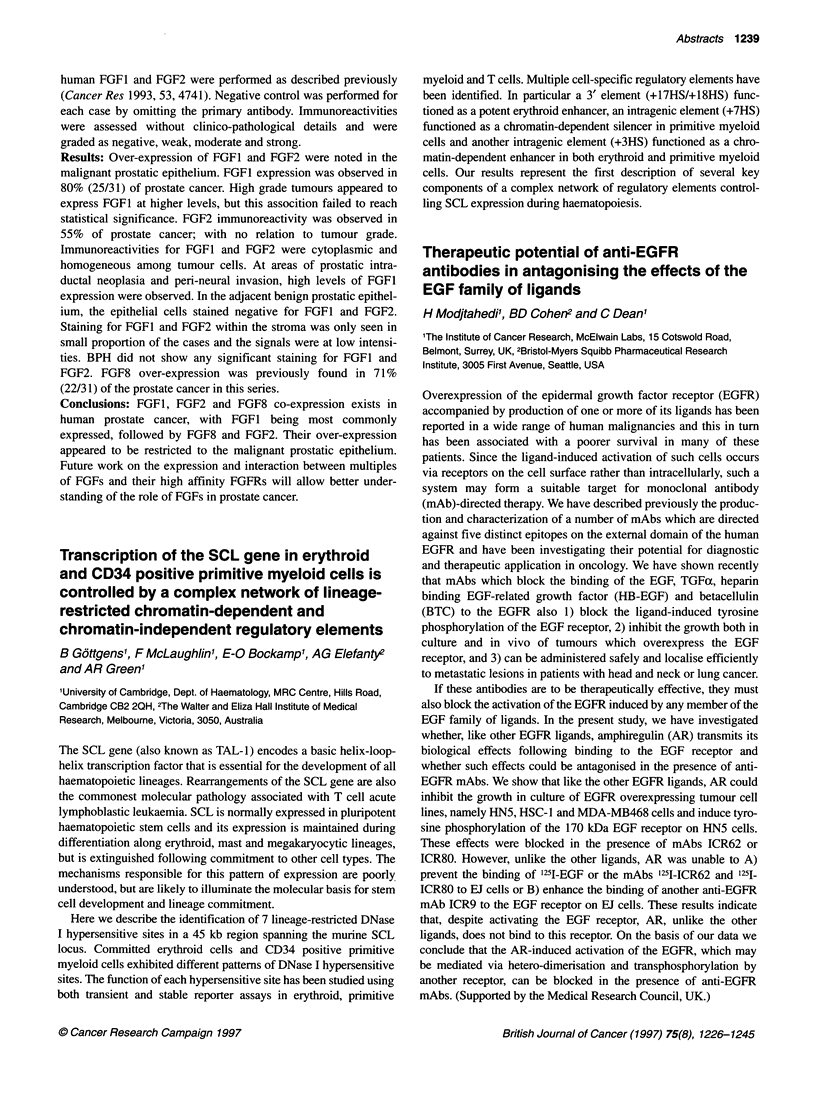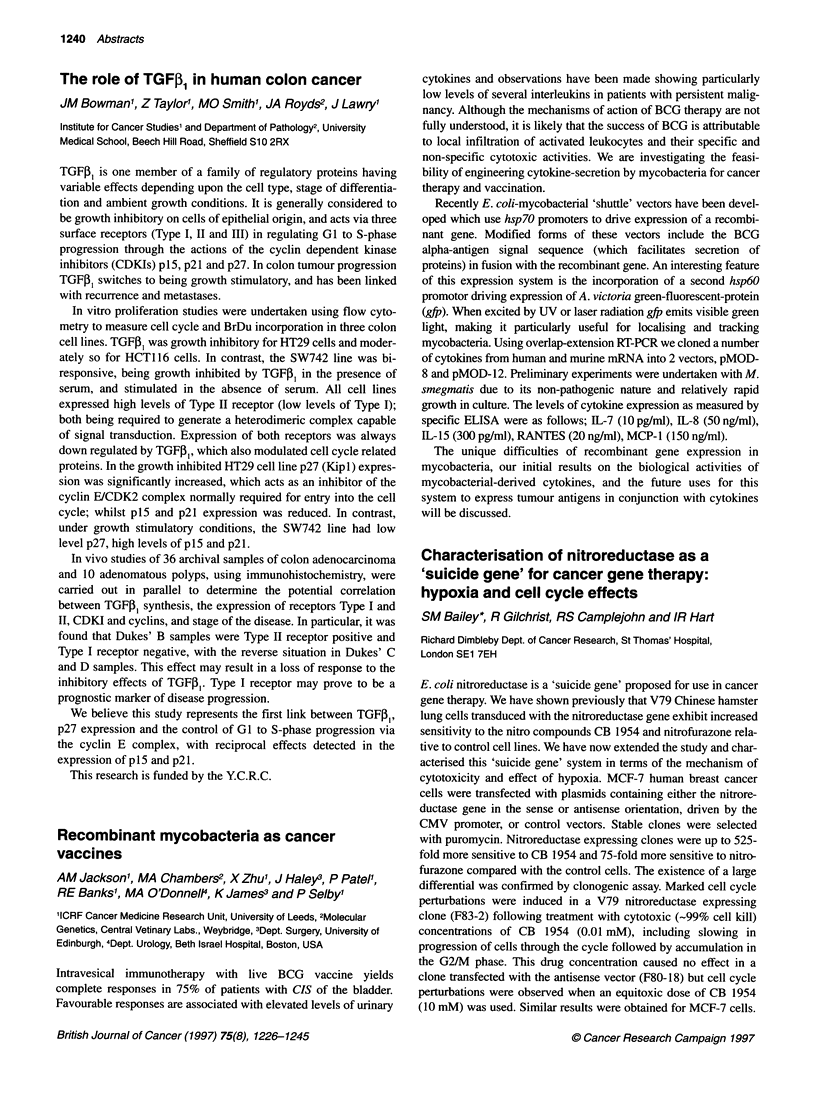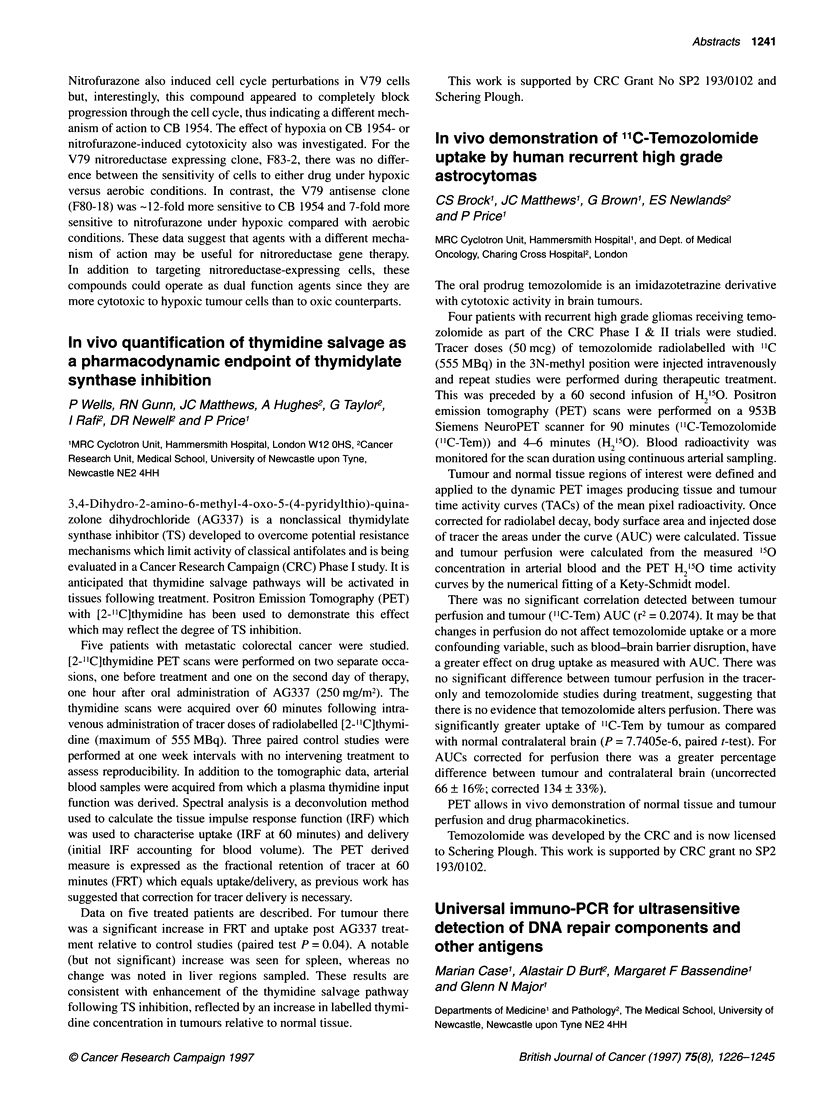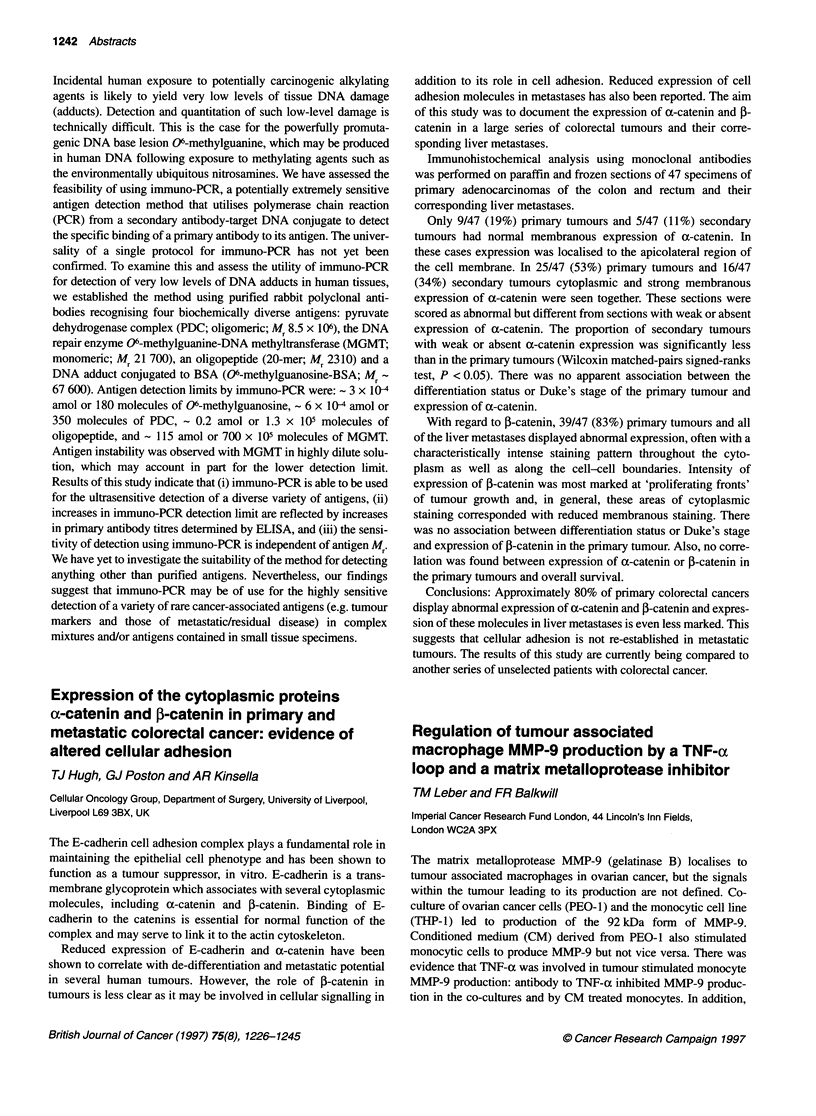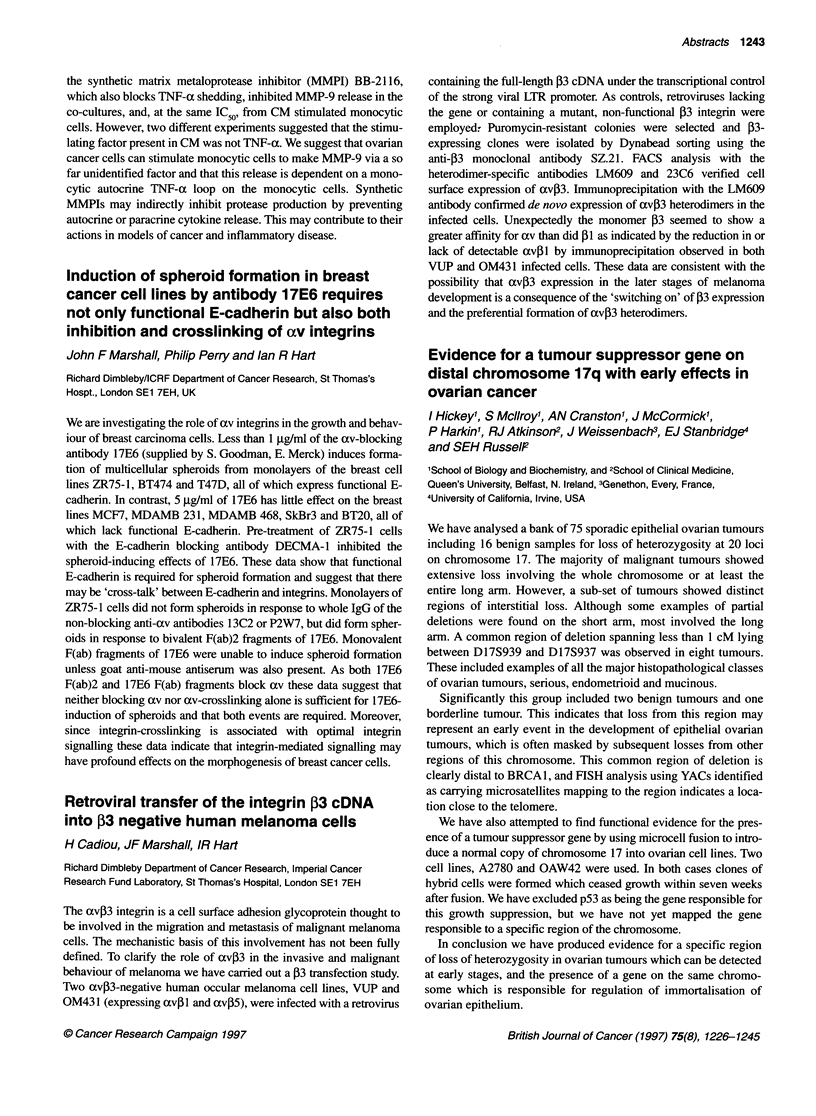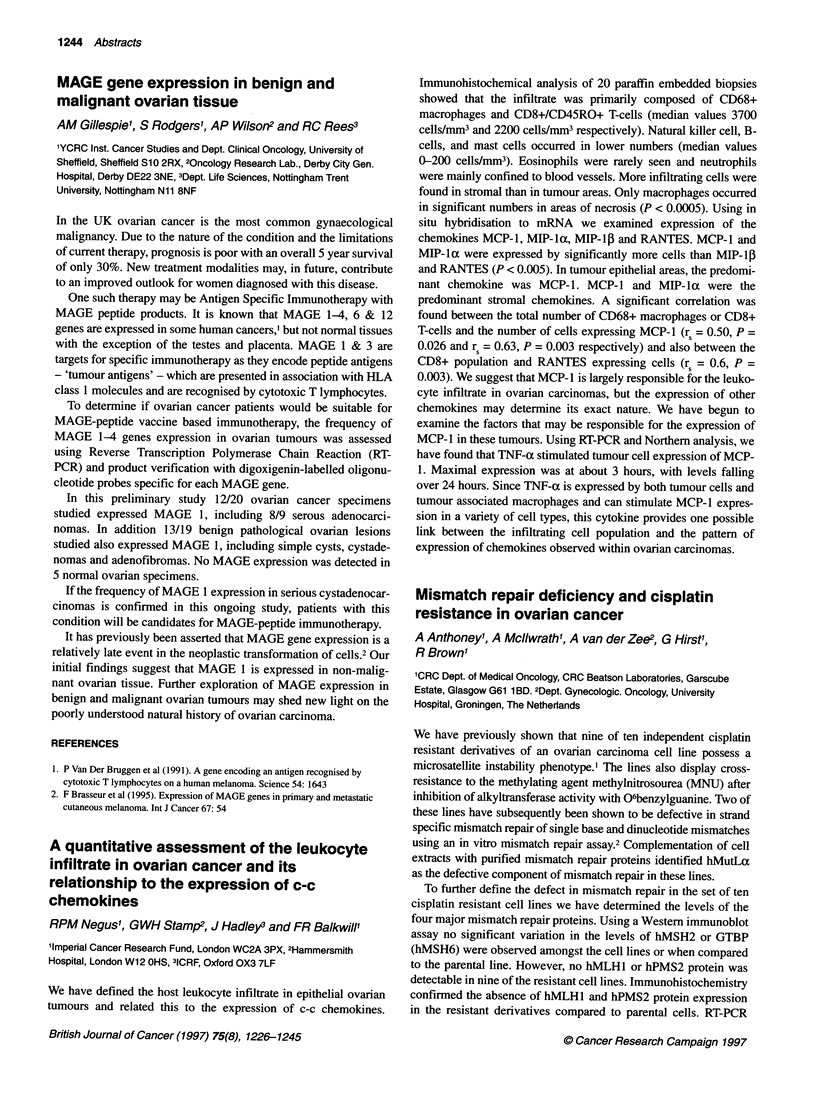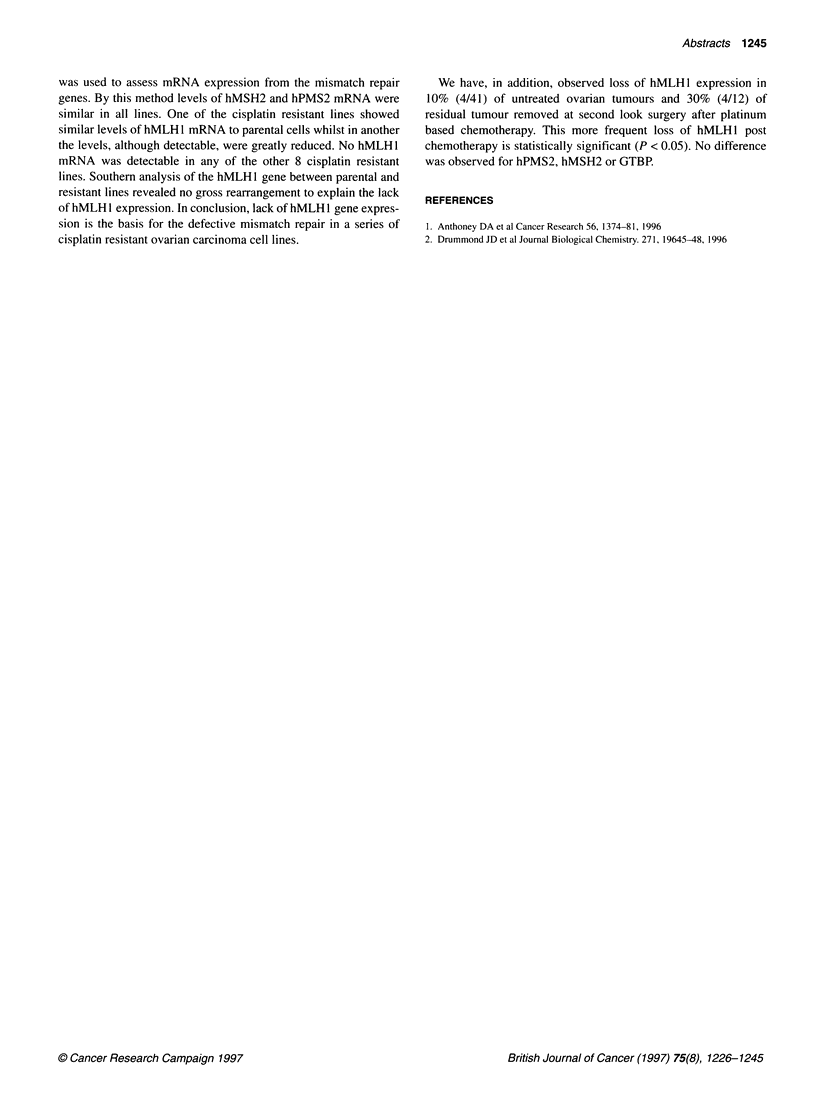# Molecular pathology of cancer

**Published:** 1997

**Authors:** 


British Journal of Cancer (1997) 75(8), 1226–1245
© 1997 Cancer Research Campaign

Abstracts

### Molecular pathology of cancer

British Association for Cancer Research Association of Cancer Physicians and Royal Society of Medicine (Oncology Section): Joint Winter Meeting, 2–3 December 1996, Royal Society of Medicine, 1 Wimpole Street, London W1, UK

### PCR based diagnosis of micrometastatic and residual cancer

*P Selby*

ICRF Cancer Medicine Research Unit, St James's University Hospital, Leeds LS9 7TF

PCR has provided an opportunity to detect small quantities of cancer cells. This has been applied to the detection of micrometastatic and microresidual cancers in the blood and bone marrow. Haematological cancers may present consistent abnormalities of DNA with characteristic translocations which provide targets for detection. Successful detection of micrometastatic disease using these approaches is now common for several haematological cancers although the complexities of interpretation of the data must be emphasised. Technical problems are common and the extreme sensitivity of the technique may reveal translocations to be present at a low level in normal tissues. Solid tumours represent a greater technological challenge. Specific abnormalities of DNA are less well described. An alternative approach has been to detect the expression of genes which are specific to the tissue of origin of a cancer but are not normally expressed in the tissue under study for the presence of micrometastatic disease, usually blood or bone marrow. This approach has proved promising for a number of cancers, particularly melanoma, neuroblastoma, prostatic cancer, with some less certain success for breast and colorectal cancer. The clinical situations where useful information may be found include the detection of micrometastatic disease as part of staging, during and after treatment in follow-up and the detection of contamination of peripheral blood stem cell harvests. Data suggest that we should investigate the role of these techniques but the technical challenges are considerable and comparisons between interested laboratories have shown a persisting difficulty with false positive and negative tests.

### Minimal residual disease in solid cancer: chance and challenge for new diagnostic and therapeutic strategies

*G Riethmuller*

Institute of Immunology, University of Munich, D-80336, Munich

Despite progress in early diagnosis metastasis frequently precedes detection of primary tumours in patients with the most common types of epithelial cancer. Thus, systemic and occult dissemination of cancer cells accounts for a sizeable proportion of cancer mortality in the Western industrialized countries. Recent advances in immunological and molecularbiological techniques have made possible the detection of isolated disseminated tumour cells. In various tumours, the presence of such cells in bone marrow has been shown to have prognostic significance for the clinical development of the disease, i.e. for a manifest relapse at distant sites. Because of their relatively low number and accessible location in mesenchymal compartments isolated metastatic cells are ideal targets for new biological or immunological therapeutic strategies. Immunotherapies relying on cooperative activities with the patient's immune system appear particularly attractive in the early stage of minimal residual disease (MRD). Diagnostic and therapeutic data on MRD of various solid tumours will be presented in my presentation.

### Solid phase separation techniques have a role in the detection of rare tumour cells in either peripheral blood or bone marrow

*JT Kemshead*

Oncology Laboratories, Frenchay Hospital, Bristol BS16 1LE

Conflicting claims have been made in the literature regarding the level at which tumour cells can be detected in peripheral blood and bone marrow by either immunological or molecular techniques. Our experience with respect to the detection of neuroblasts in blood products will be reviewed, with particular relevance to the routine use of these procedures in a clinical setting. However, irrespective of the methodology, the sensitivity of tumour cell detection is limited by the volume of the sample which can be processed. This limitation can be avoided if a pre-enrichment procedure is used to partially enrich the tumour population. A strategy to develop a solid phase separation procedure where rare tumour cells can be isolated onto a solid phase matrix and subsequently released will be discussed. This technology is based upon the use of an immunomagnetic colloid and genetically engineered antibodies.

Despite the advances made in detecting metastatic tumour cells in blood and bone marrow, the clinical relevance of this work is hotly debated. Using gene marking techniques it has been demonstrated that, in children with neuroblastoma, contaminated blood products can contribute to the relapse of patients. These studies have been undertaken in children receiving autologous marrow as a rescue procedure after high dose consolidation therapy. This data proves that such grafts should be subjected to 'purging techniques' to ensure that they are free of tumour cells. However, some groups still procrastinate about the use of this technology as they argue it is unnecessary until consolidation therapies become more effective. Other centres continue to the drive to ensure that the tumour burden is reduced to a minimum and consequently pursue the development of highly sensitive purging techniques. We have piloted such a procedure whereby CD34 positive cells are initially isolated from either bone marrow or peripheral blood stem cell grafts. This population is then subjected to a 'tumour purge' using a cocktail of monoclonal antibodies and a solid phase separation technique. Calculations indicate that this procedure can essentially remove all tumour cells from either marrow or peripheral blood stem cell harvests and experimental data confirms both its sensitivity and safety.

### Minimal residual disease in the leukaemias

*John M Goldman*

Haematology Department, Royal Postgraduate Medical School, London, UK

Residual leukaemia after treatment has in the past been defined at the morphological or cytogenetic levels. In the last five years molecular methods, especially RT-PCR, have been developed and tested in patients in seeming complete remission after treatment for acute and chronic leukaemias. Chronic myeloid leukaemia (CML) is probably the prototypic disease in which RT-PCR for BCR-ABL transcripts has proved clinically useful. CML patients treated by allogeneic BMT with unmanipulated donor marrow often have molecular evidence of residual disease for 6–9 months post-transplant but this may disappear thereafter; conversely, patients who receive T-depleted donor marrow may remain PCR-positive for long periods post-BMT. Relapse may be defined on the basis of rising numbers or persistently high numbers of transcripts more than 9 months post-BMT and is presumably due to relative failure of a GVL effect. Treatment may be instituted with low doses of donor lymphocytes which re-induce remission in 70–80% of cases. CML patients treated with interferon-α who achieve complete cytogenetic remission remain PCR-positive with rare exceptions but the numbers of transcripts in different patients spans 4 orders of magnitude. Ph-negative 'high' PCR-positive patients may be candidates for auto-harvesting and autotransplants in remission; 'low' PCR-positive patients include some who may be regarded as operationally 'cured' and may need only 'observation'. The pattern is somewhat less clearcut in AML. AML-M3 is characterised by a PML-RARα fusion gene. Detection of the transcript in chemotherapy-treated patients is associated with a high risk of relapse, whereas PCR-negative patients have a low risk of relapse. The sensitivity of the assay may be increased by studying the reciprocal fusion transcript, RARα-PML. Conversely, AML patients with t(8;21) have a chimeric mRNA for the AML1-ETO fusion gene but its detection in remission does not predict relapse. This suggests that this fusion gene may not be the sole pathogenetic mechanism operating in this sub-type of leukaemia. Preliminary data also suggest that detection of the chimeric transcript for the CBFβ-MYH11 fusion gene characteristic of AML with inv[16] also correlates poorly with the risk of relapse. Residual disease after treatment for ALL may be monitored by specific chimeric transcripts or clonal rearrangements of IGH or TCR genes; a potentially valuable approach is based on the observation that 20–25% of children with ALL have a TEL-AML1 fusion gene; corresponding transcripts are undetectable after treatment in some patients. In summary, molecular methods for following patients with leukaemia in apparent remission seem to be of increasing value and will in the coming decade constitute an important basis for 'tailored' therapy.

### Genetics of the malignant progression of human brain tumours

*W Cavenee*

Ludwig Institute for Cancer Research, Dept. of Medicine and Center for Molecular Genetics, Univ. California-San Diego, La Jolla 92093–0660

One model for the malignant progression exhibited by human cancer invokes clonal evolution and selection of population subsets with increasing deviance from the norm. We have been particularly interested in the suitability of the model to astrocytic brain cancer. Low grade glial tumours are distinguished phenotypically from normal brain by their greater degree of cellularity, nuclear atypia and increased microvessel density. From a genetic standpoint, mutation of the p53 gene is the earliest detectable genetic lesion and by analysing its mutations in low grade and corresponding recurrent high grade tumours, direct support has been derived for the clonal evolution model. Wild-type p53 but not common tumour mutants causes cessation of growth following dramatic morphological changes. Thus, the cell number and shape changes seen in low grade disease may be attributable to this early genetic lesion. Further evidence was obtained by deriving cultures of primary cortical astrocytes from mice whose endogenous p53 alleles were genetically 'knocked out'. These studies showed that immortalization was conferred by p53 allele inactivation and that this process engendered an increased probability of cell transformation. Wild-type p53, but not its commonly occurring mutant forms, caused glioma cells to secrete an inhibitor active in limiting both endothelial cell migration and neovascularization. This inhibitor neutralized the positive angiogenic factors secreted by the tumour cells, such as bFGF or VEGF. In the case of the latter factor, expression of antisense constructs resulted in ablation of tumourigenicity.

Finally, we have identified additional genetic lesions in high grade disease. These include alterations in one component or another of the p16/CDK4/CDK6/RB complex required for control of the G1 cell cycle checkpoint as well as truncation and amplification of the EGF receptor. In the latter case, molecular dissection of the signal transduction pathways has led to the identification of a specific inhibitor which may be useful as an adjunct to traditional therapies.

### Identification of initiating genetic events in human neoplasia

*D Wynford-Thomas*

Dept. Pathology, UWCM, Cardiff, UK

Although most human tumours can now be associated with multiple somatic mutations, identifying the key initial event is still

© Cancer Research Campaign 1997 

problematic. Presence of the genetic abnormality in the earliest recognisable tumour stage is a necessary criterion (and sometimes crucial in preventing misleading extrapolation from experimental models) but even in 'multi-stage' tumours can never exclude prior cryptic events. Proof of causality depends on demonstration that the genetic lesion is sufficient to induce an experimental analogue of the early tumour phenotype, for which the most popular approach is to use tissue-specific expression in a transgenic mouse. While this is undoubtedly a valuable model for familial cancers, expression of a mutant gene in all cells of a tissue and from an early developmental stage is a poor model of somatic mutation arising in a single adult cell, and not surprisingly can generate misleading pathologies, particularly with activated oncogenes. Our laboratory has used the complementary approach of gene transfer into normal epithelial cells in vitro, which allows use of human cells (although of course has limitations imposed by tissue culture). Using amphotropic retroviral vectors the earliest stages in development of tumours of the thyroid follicular cell were successfully reconstructed, and provided strong evidence that the resulting phenotype ('follicular' or 'papillary') is determined by the 'choice' of initiating oncogene (ras or ret respectively), thus providing a molecular explanation for the occurrence of divergent tumour pathologies from a common cell of origin. This model has also been exploited to develop novel approaches to early therapy or prevention, based on targetting the signal pathways of the initiating oncogene. Although the picture is still incomplete, transgenic and gene transfer models of different tumour types are beginning to reveal relationships between the nature of the initiating event and the cell kinetics of the normal tissue, e.g. activation of oncogenes in mitotically-inactive thyroid, contrasting with inactivation of a tumour suppressor gene in renewing epithelium of colon. Such data may provide new insights into the tissue-specific rate-limiting controls of normal cell proliferation.

### Genetic control of tumour predisposition and progression in mice

*A Balmain*

CRC Beatson Laboratories, Department of Medical Oncology, University of Glasgow, Garscube Estate, Bearsden, Glasgow G61 1BD

Tumours develop in both mice and humans as a consequence of several genetic alterations at critical genomic loci. These changes involve mutations in oncogenes such as *ras*, and in tumour suppressor loci, including the p53 and p16 genes. Our strategy has been to investigate the genetic alterations which take place during mouse skin tumour development, and subsequently to use both transgenic and knock-out mice to test the functions of candidate genes in vivo. Genes implicated in multistage carcinogenesis include H-*ras*, p53 and TGFβ. We have generated transgenic lines expressing H-*ras* and TGFβ in specific subpopulations of epidermal cells using keratin gene promoters. These animals show alterations in epidermal growth and differentiation and in their susceptibility to tumour development. Mice expressing TGFβ develop fewer papillomas than non-transgenic littermates, but have elevated rates of tumour progression. This indicates that TGFβ can act either positively or negatively at different stages of carcinogenesis. The p53 tumour suppressor gene plays a critical role in prevention of tumour progression, by mediating apoptosis in response to accumulating genetic damage. Mice deficient in p53 show reduced apoptosis, and consequently an increased progression frequency.

Another class of genes which are important in tumour development are those which control genetic predisposition. Different mouse strains have differing susceptibilities to develop either benign or malignant tumours, and several of these genes have been mapped in crosses between sensitive and resistant strains. Many of these germline predisposition loci show alterations in somatic cells during tumour progression, suggesting that they act as bona fide tumour suppressor genes.

### The identification and characterisation of BRCA2

*M Stratton*

Section of Molecular Carcinogenesis, Institute of Cancer Research, Sutton, Surrey SM2 5NG

The breast cancer susceptibility gene, BRCA1, was located on chromosome 17q in 1990. Subsequently an analysis of over 200 families with multiple cases of breast cancer suggested that although the large majority of families with both breast and ovarian cancer are due to this gene, only 50% of families with site specific breast cancer are due to BRCA1. The existence of a further susceptibility gene was confirmed by an analysis of families with at least one case of male breast cancer (and usually several of early onset female breast cancer), most of which appear not to be due to BRCA1. A genome search resulted in the localisation of BRCA2 to chromosome 13q12-q13 in 1994.

Evidence from breast cancers arising in mutation carriers provided evidence that BRCA2 is a tumour suppressor gene and loss of heterozygosity on chromosome 13q was found in approximately one third of sporadic breast cancers. BRCA2 was isolated in 1995 by positional cloning. The gene encodes a large predicted protein of 3,318 amino acids that bears no substantial sequence similarity to other proteins in the publicly accessible databases although there is an eight times repeated 25 amino acid motif of an unknown function. Germline mutations in BRCA2 usually result in truncation of the protein and hence likely inactivation of critical functions. Somatic mutations of BRCA2 in sporadic breast or ovarian cancers have not been found. Mutations in BRCA2 are now believed to result in an excess risk of ovarian cancer, leukaemia, prostate cancer, pancreatic cancer, and endometrial cancer in addition to male and female breast cancer.

### Genetic analysis of early breast lesions

*JM Varley, K Marsh, K Munn and L James*

CRC Department of Cancer Genetics, Paterson Institute for Cancer Research, Wilmslow Road, Manchester M20 9BX

Ductal carcinoma in situ of the breast (DCIS) is considered by many to be a preinvasive precursor to frankly invasive tumours. We have analysed a number of cases of DCIS which are either 'pure' DCIS or associated with invasive tumours, for LOH at a number of chromosomal loci. For the analysis, multiple independent ducts are

 *© Cancer Research Campaign 1997*

microdissected from formalin-fixed, paraffin-embedded sections, and each duct is analysed separately. If present, regions of invasive carcinoma are similarly prepared from the same section. In all cases sections of normal breast are available as a control. We have concentrated on regions of the genome known to show high levels of LOH in invasive tumours, including regions of chromosomes 1, 9 and 17. All these regions show loss at levels which are comparable to those seen in invasive tumours, and furthermore are present in well-differentiated lesions as well as poorly-differentiated DCIS. We have seen considerable levels of heterogeneity between ducts of the same subtype, as well as between ducts of different subtypes.

Comparative genome hybridisation (CGH) is a technique which allows the genome-wide examination of regions of chromosomal loss or gain using techniques of fluorescence in situ hybridisation. A number of cases of DCIS have been analysed using this technique, after microdissection and pooling of multiple lesions from the same patient. A wide variety of abnormalities were detected, including gain and loss of material, and high-level amplification of some areas was also observed. Chromosomal alterations are more frequent in higher grade DCIS, and closely resemble those previously detected in invasive breast cancer using the same technique.

Taken together, our data indicate that most, if not all, the genetic alterations seen in invasive breast tumours are present in DCIS. We have seen loss and gain of sequences in lesions of all grades and degrees of differentiation, although the changes are more numerous and more frequent in DCIS lesions of high nuclear grade and poor differentiation.

### Genetic events in the development and progression of bladder cancer

*Margaret A Knowles*

Molecular Genetics Laboratory, Marie Curie Research Institute, The Chart, Oxted, Surrey RH8 0TL

Bladder cancer is a heterogeneous disease. Two major groups of tumours exist (superficial papillary and invasive) with very different clinical outcome. It is anticipated that a detailed knowledge of the molecular genetics of these lesions will provide a basis for understanding this divergent clinical behaviour and should generate useful tools for diagnosis, prediction of prognosis and ultimately for therapeutic intervention.

Many common genetic changes have been identified in transitional cell carcinoma (TCC) of the bladder. These include alterations to proto-oncogenes (HRAS, ERBB2 and CCND1), mutation or homozygous deletion of tumour suppressor genes (TP53, RB1 and CDKN2 [p16]) and frequent loss of heterozygosity (LOH) of chromosome arms 3p, 4p, 4q, 8p, 9q, 11p, 11q and 14q. Detailed mapping and positional cloning projects are underway to identify candidate genes within regions of frequent LOH, particularly those on the long arm of chromosome 9 which is deleted in the majority of bladder tumours of all grades and stages. We have mapped the common regions of LOH on chromosomes 4p, 8p and 9q in detail. Two critical regions on 4p, at least 2 regions on 8p and 3 regions on 9q have been defined. Precise localisation for two putative target loci have been obtained and analysis of candidate genes from these regions will be presented.

Until recently, the natural history of development of papillary and invasive TCC was not well defined. Molecular genetic findings now indicate that two major pathways of progression exist, one via carcinoma in situ ->invasive TCC, the other via hyperplasia ->dysplasia->papillary TCC. The temporal ordering of events in these pathways is not clear though LOH of chromosome 9 is considered a likely 'early' event. Thus, identification of the relevant tumour suppressor genes on chromosome 9 is considered pivotal to progress in understanding the development and clinical behaviour of TCC.

### Heparan sulphate and the development of oligosaccharide growth factor inhibitors

*GC Jayson[1], C Paraskeva[2], JT Gallagher[1]*

[1]CRC Dept. of Medical Oncology, Paterson Institute for Cancer Research, Withington, Manchester M20 4BX. [2]Dept. CRC Colorectal tumour biology group, Dept. Pathology and Microbiology, Univ. of Bristol, Bristol BS8 1TD

Heparan sulphate (HS) is a widespread, complex, linear, cell surface and extracellular matrix glycosaminoglycan that is of fundamental importance in the control of cell growth. HS is of increasing clinical interest because it acts in a catalytic fashion to increase the binding and activation of a number of growth factors, including FGF-1, -2, -4, VEGF and hepatocyte growth factor (HGF). These observations have focused attention on HS as a target for new anti-cancer strategies. To determine the optimum method of targeting HS we analysed the structure of HS derived from the first in vitro model of the progression from human adenoma to carcinoma. The transition to a carcinomatous phenotype was associated with a 50% reduction in the synthesis of HS relative to CS and a significant reduction of syndecan 1 and 4 mRNA. In HS specific structural changes were seen during progression, including a 17% reduction in total sulphation, a 22% reduction in N-sulphation, a 50% reduction in 2-O-sulphation and a 17% increase in 6-O-sulphation. The chains were trisected by two sulphated domains that were larger in the adenoma HS. On average an adenoma HS chain contained 2 mono- and 3 di-sulphated nitrous acid resistant tetrasaccharides, while a carcinoma HS chain contained 1 mono-, 2 di- and 1 tri-sulphated nitrous acid resistant tetrasaccharides.

Basic FGF was an effective mitogen for the adenoma cells ($EC_{50}$ = 1 ng/ml) but not for the carcinoma cells, yet RT-PCR techniques detected the same classes of FGF-R in the adenoma and carcinoma cells. Investigation of the relationship between adenoma HS and bFGF and carcinoma HS and bFGF showed that progression is associated with a change in dissociation constant from 220 nM to 2493 nM, suggesting that HS may in part control the cells' response to bFGF.

HS undergoes specific structural and functional changes during progression, implying that it is a potential target for new anti-cancer strategies. In further experiments Caco-2, a human colon carcinoma cell line, was adapted to growth in serum free medium. This model responds to bFGF with an increase in cell multiplication and movement and the mitogenic effect of bFGF is dependent on HS. The ability of heparin oligosaccharides of different lengths but uniform composition to inhibit these activities was tested. This showed that the optimum length of an inhibitory oligosaccharide was an octasaccharide which caused a significant reduction in both bFGF induced mitogenesis and motility at 10μg/ml, a clinically achievable concentration.

© Cancer Research Campaign 1997

### Histotypes, clonality and cytotoxic molecules in Hodgkin's disease

*H Stein, M Hummel, T Marafioti, H-D Foss*

Institute of Pathology, University Hospital Benjamin Franklin, Freie Universitat, 12200 Berlin, Germany

By the demonstration of a constant expression of the lymphoid cell activation associated cytokine receptor CD30 and an occasional expression of B- or T-cell antigens on Reed-Sternberg cells we had provided growing evidence suggesting that Hodgkin's disease can be B-cell related or T-cell related. To further elucidate this issue we and others analysed single Reed-Sternberg cells for rearranged Ig variable (V) genes and cytotoxic molecules (granzyme B and perforin). For the detection of IgV rearrangements single Reed-Sternberg cells were isolated from frozen sections that had been immunostained for CD30 and analysed by PCR followed by sequencing. The cytotoxic molecules were either demonstrated by immunohistology (granzyme B) or by in-situ hybridization (perforin). Using various primer sets we found identical IgV rearrangements in Reed-Sternberg cells in eight and unrelated IgV rearrangements in two of ten B-cell immunophenotyped HD cases. Cytotoxic molecules were demonstrated in Hodgkin's disease with a T-cell or null-cell immunophenotype accounting for 10–20% of all Hodgkin's disease cases. These findings confirm the existence of Hodgkin's disease cases with B-cell derived Reed-Sternberg cells and Hodgkin's disease cases with T-cell derived Reed-Sternberg cells. An EBV infection could be demonstrated in both B-type and T-type Reed-Sternberg cells.

### Prognostic factors in cancer: myths, mistakes, and the Holy Grail

*Peter A Hall[1] and Neil A Shepherd[2]*

[1]Dept of Molecular and Cellular Pathology, University of Dundee DD1 9SY and [2]Dept of Cellular Pathology, Gloucestershire Royal Hospital, Gloucester GL1 3NN

The search for parameters that might facilitate the prediction of patient outcome is a highly prevalent activity in oncology: indeed it represents a form of Holy Grail. During the period from January 1993 to July 1996, 2531 publications are listed by BIDS as having the term **prognosis** in the title and 16 078 with the same word in title and abstract. Examination of an unselected series of these publications in mainstream oncology, pathology or generalist journals indicate that many studies are methodologically flawed, frequently being based upon small numbers of patients (with consequent small power) and being poorly controlled. The use of more than one data set is vanishingly rare. Prospective studies to test analyses do not predominate and the phrase '... this parameter may be of prognostic significance' is sadly common. The cut-off points employed are rarely validated and often have no clearly defined biological rationale. While a significant subset of studies endeavour to employ multi-parameter (usually Cox) modelling many fail to make comparisons with commonly used and well established prognostic parameters. Most studies are performed in the context of tertiary referral or other specialist centres and the applicability of results to the 'real' world is not often clear.

While there is no question that the aims are valid and worthwhile this paper presents a critical analysis of prognostic studies and urges the use of more rigorous and scientific approaches including the application of multiple data sets, the use of fully unselected series, the experimental testing of cut-off points and the more realistic consideration of the long term practicability of methods in real clinical settings.

### The tumour suppressor gene p53 as a predictive factor in human breast cancer

*J Bergh*

Dept of Oncology, Univ. of Uppsala, Akademiska sjukhuset, S-751 85 Uppsala, Sweden

**Introduction:** p53 is the conductor for many essential cellular processes, e.g. control of proliferation, differentiation and apoptosis. The gene is localised to band 13.1 on the short arm of chromosome 17. The protein consists of 393 amino acids with a DNA binding region between amino acids 102 and 292. Preclinical studies have demonstrated that certain cytostatics, tamoxifen and radiation may induce programmed cell death via the p53 pathway. Previous clinical studies have revealed prognostic importance for p53 for a wide range of malignant tumours. Based on these observations we started to investigate different aspects of p53, mainly with focus on its eventual predictive value in relation to used oncological therapies for breast cancer.

**Material and methods:** We isolated tumour cDNA and sequenced all exons from p53 obtained from a population and consecutive cohort of 316 breast cancer patients operated between 1987 to 1989. We also studied immunohistochemical determination of p53 using the monoclonal antibody Pab 1801 compared with sequencing. We have a median follow-up of 57 months and a maximum of 87 months.

**Results:** 69 mutations were found, 29 in the node positive group. The mutation sites were partly dissimilar between the node positive and node negative group. Node positive patients tended to have relatively more mutations within the evolutionary conserved regions II and V which was associated with very grim prognosis. Lymph node positive patients with p53 mutations treated with loco-regional radiotherapy and tamoxifen had a significantly worse prognosis compared with those without p53 mutations. Vice versa, lymph node negative patients with p53 mutations postoperatively treated with radiotherapy had a significant survival benefit while those patients without p53 mutations had no major effect on local recurrence and survival. The discriminatory clinical findings between the node positive and negative patients with respect to p53 may be due to different mutation sites leading to dissimilar functional implications for the p53 protein. The prognostic information was better using sequencing and immunohistochemistry failed to detect all 6 stop codons, 11/13 deletions and 1/3 insertions.

**Conclusion:** Our studies strongly indicate the predictive value of p53 in relation to different adjuvant therapy modalities with different implications for node positive and negative breast cancer patients, which may be related to partly different sites of the mutations.

### REFERENCES

1. Bergh J et al Nature Medicine 1:1029–1034, 1995
2. Jansson T et al J Clin Oncol 13:2745–2751, 1995
3. Sjögren S et al J Natl Cancer Inst 88:173–182, 1996

© Cancer Research Campaign 1997

### Molecular analysis and prognosis in colorectal tumours

*AH Wyllie, VJ Bubb, RG Morris, L Curtis, M Potter, Sir Alastair Currie*

CRC Labs., Dept. of Pathology, University Medical School, Edinburgh EH8 9AG

Molecular analysis of human colorectal carcinomas reveals two major sub-types. In the first, there is defective p53 function throughout the carcinoma, apparently involving the founder cells of the malignant clone. Carcinomas of this sub-type – which is responsible for around 70% of all colorectal cancers – have aneuploid karyotypes and are associated with multiple chromosome deletions and amplifications. The second major sub-type (responsible for around 15% of all) is characterised by micro-satellite instability, probably arising through deficient mis-match repair functions. These tumours are usually near-diploid, tend to be right-sided, commonly display mucinous or poorly differentiated histology and have a conspicuously better prognosis than tumours of the first sub-type. A small number of cancers appear to possess qualities intermediate between these sub-types. The questions arise whether these malignant tumours evolve from pre-existing adenomas by similar or different pathways, and whether patients bearing such tumours can be recognised at an early stage.

### The role of p53 and related mediators and effectors of p53 function in the molecular pathology and prognosis of cancer

*J Lunec*

Cancer Research Unit, Univ of Newcastle upon Tyne Medical School, NE2 4HH

The p53 tumour supressor gene is frequently found to be mutated in a wide range of common sporadic tumours. Gene transfer in vitro and in vivo as well as germline inactivation of the p53 gene in mice have established clear causal links for the tumour suppressor role of p53. The p53 gene encodes a nuclear phosphoprotein that binds to DNA in a sequence specific manner and acts as a transcription factor for the regulation of genes that play key roles in cell cycle progression and in the response of cells to DNA damage. The cellular levels of p53 protein accumulate rapidly in response to DNA damage and other cellular stresses. If the p53 is normal, this leads to either growth arrest or apoptotic death, depending on the cell type and the level of damage. Thus the functional p53 status of cells is likely to be an important determinant of tumour progression and response to treatment. The role of p53 and potential prognostic significance will be illustrated by data on bladder and ovarian carcinoma and contrasted with the different behaviour in childhood neural tumours.

Mutations of the p53 gene appear to be a late step in the progression of bladder cancer, since they are found infrequently in superficial tumours, but are detected at a high rate in late stage high grade tumours. Patients with late stage (T2–T4) tumours with detectable p53 mutations have a trend towards worse prognosis, with our own studies showing a median survival ratio of 3.54–4.46 (95% confidence interval) for normal compared with mutant p53 cases. For superficial tumours, a link between p53 mutations and a higher risk of progression has been reported, but the results are equivocal and larger studies are required. In ovarian cancer p53 mutation is more frequent in late stage poorly differentiated tumours and complete response to pharmacologically defined single agent platinum-based chemotherapy is strongly associated with the absence of detectable p53 mutations.

In bladder and ovarian cancer mutational inactivation of p53 is frequently associated with abnormal p53 accumulation. However, in a significant proportion of cases abnormal accumulation of wild-type p53 protein occurs and this raises questions about the functional integrity of p53 pathways in these tumours. In tumours of the peripheral and central nervous system p53 staining in the absence of mutations predominates and in neuroblastoma cytoplasmic sequestration of p53 has been reported. As understanding of the mediators and modulators of p53 function has developed, increasing attention is being devoted to assessment of the functional integrity of p53 related pathways rather than the mutational status of p53 alone. New information on the role of WAF1 and *mdm*2 expression in bladder and ovarian cancer will be discussed.

### Genetic alterations in in situ carcinoma and putative precancerous lesions of the breast

*SR Lakhani[1], N Collins[2], MR Stratton[2] and JP Sloane[3]*

[1]Dept of Histopathology, UCLMS London WC1E 6JJ, [2]Section of Molecular Carcinogenesis, ICR, Surrey SM2 5PT and [3]Dept of Histopathology, Royal Liverpool University Hospital, Liverpool L69 3BX

Ductal carcinoma in situ (DCIS) and borderline lesions are being encountered with increasing frequency since the introduction of mammographic screening. Low Nuclear Grade variants of DCIS are difficult to distinguish from Lobular Carcinoma in situ (LCIS), Atypical Ductal Hyperplasia (ADH) and florid Hyperplasia of Usual Type (HUT). Unlike DCIS, LCIS is more often multifocal and bilateral and invasive carcinoma subsequent to LCIS is often of the ductal phenotype. Hence, there is doubt as to whether LCIS is hyperplastic or neoplastic and whether it is a precursor lesion or simply a marker of risk. Although considered benign, ADH is associated with a significant risk of developing invasive carcinoma. These considerations raise interesting questions about the biological nature and interrelationships of these lesions.

Using a microdissection technique, polymorphic microsatellite markers and the polymerase chain reaction (PCR), loss of heterozygosity (LOH), at loci known to be present in high frequency in invasive breast carcinoma (IC), was investigated in in situ carcinoma and other proliferative lesions of the breast. 132 cases of DCIS, 43 cases of LCIS, 14 cases of ADH and 51 cases of HUT were examined for LOH on chromosomes 16q, 17p, 17q and 13q.

DCIS and LCIS showed a similar frequency of LOH to IC at the loci studied (range 25–55%) apart from a locus on 17p (in the vicinity of TP53) where the frequency of LOH in LCIS was much lower (8% in LCIS vs 39% in DCIS). ADH exhibited a similar frequency of LOH to DCIS and IC. HUT, however, exhibited LOH in only a small proportion of cases (range 0–13%). There were no morphological or cytological features in ADH or HUT that predicted LOH.

The findings indicate that like DCIS, LCIS is a neoplastic proliferation and likely to be a direct precursor of invasive carcinoma.

© Cancer Research Campaign 1997

The difference in LOH at 17p in LCIS suggests that the morphological and clinical differences between LCIS and DCIS are also reflected at the genetic level. The similar incidence at other loci, however, suggests that they may share a common pathway of evolution. The evidence shows that ADH is a neoplastic rather than a hyperplastic proliferation as its name implies and perhaps best considered within the spectrum of ductal in situ neoplasia. At least a proportion of HUT are also clonal and hence represent benign neoplastic proliferations (adenomas) of the breast epithelium. Hence these lesions are likely to represent non-obligate precursors of invasive carcinoma.

### Investigation of the progression of breast cancer from ductal carcinoma in situ (DCIS) to invasive carcinoma by comparative genomic hybridisation (CGH)

*E Moore[1], H Magee[1], M Murphy[1], J Coyne[2], PA Dervan[1]*

[1]Department of Pathology, Biotechnology Centre, University College Dublin and [2]Withington Hospital, Manchester

Progression of a tumour is presumed to involve a series of genetic aberrations which confer increasing growth factor independence to somatic cells. A novel technique, comparative genomic hybridisation (CGH)[1], is employed in which the relative intensities of tumour DNA (detected with green fluorescence) and normal reference DNA (detected with red fluorescence) after hybridisation to normal metaphase chromosomes is used to reveal and map changes in DNA-sequence copy number. This approach has been applied to the investigation of DNA copy number changes involved in the progression of breast cancer from the earliest recognisable stage, DCIS to invasive carcinoma. In this initial study CGH was applied to 10 primary carcinomas, 6 high, 4 intermediate and 2 low grade tumours and to 7 high, 1 intermediate and 1 low grade DCIS tumours. All carcinomas showed evidence of increased or decreased DNA-sequence copy number changes. In the invasive carcinomas the highest frequency of amplifications occured at 8q22–24, 1q21–31, 17q11–23.1 and 20q12–13 and 19q13.1, 11q24 and 1p36 showing the highest frequency of deletions. The DCIS cases revealed highest frequency of amplifications at 1q22–42, 17q, 6q11.2–26 and 13q13–32 and deletions at 16p and 17. As fresh tissue for DCIS is rarely available it was necessary to use formalin-fixed paraffin-embedded tissue for CGH analysis. To investigate a possible shift in profile due to normal and stromal cell contamination, DNA was extracted from microdissected tumour cells and used for CGH analysis following universal amplification by degenerate-oligonucleotide primed PCR (DOP-PCR). We concluded that DNA from a tumour cell population of $>50\%$ is necessary for accurate CGH analysis. These finding may provide the foundation for a future study of specific and possibly novel genes associated with the different grades of breast cancer.

### REFERENCE

1. Kallionemi OP et al Cancer Biology Vol.4, 41–46

### Expression of a BRCA1 allele containing a 12 bp insertion in intron 20

*H Russell[1], M Monaghan[1], A Woodman[1], A McKinley[1], I Hickey[2], R Atkinson[3] and N Nevin[1]*

[1]Dept. of Medical Genetics, [2]School of Biology and Biochemistry, [3]Dept. of Oncology, The Queen's University of Belfast, N. Ireland

In 1990, a breast/ovarian cancer susceptibility locus was mapped to chromosome 17q21 and the gene, BRCA1 cloned in 1994. The gene is 100 kb of genomic DNA and codes for a transcript of 7.5 kb. It is composed of 24 exons with exon 11 accounting for more than 60% of the gene. Mutation analysis shows a wide spectrum of mutations occurring throughout the gene with little evidence of mutational 'hotspots' and the majority of changes resulting in frameshift or nonsense mutations. One variant which has been described as a possible splice mutation is a 12 nucleotide duplication, 48 bp downstream of the 3' end of exon 20.

We have screened for BRCA1 mutations by SSCP and PTT in germline DNA from members of nine families with a history of early onset breast and ovarian cancer and also tumour DNA from 17 sporadic ovarian cancer patients. Four of the 17 cases have previously been shown to have partial deletions from chromosome 17 in the region of BRCA1 by loss of heterozygosity. The remainder had early onset disease, i.e. presentation before the age of 45.

An SSCP band shift with exon 20 primers was observed in the 3 affected members from one family and in tumour DNA from one sporadic cancer patient which was subsequently demonstrated in germline DNA as well. We did not detect this sequence alteration in 134 control samples which included several patients with late onset sporadic ovarian cancer as well as non-cancer controls.

We subcloned both the normal and variant alleles in these patients into a pGEMt vector and observed the 12 bp duplication 48 bp into the intron as previously described. Archival breast tumour DNA was available from one of the familial cases and LOH studies with microsatellite polymorphisms demonstrated widespread LOH from 17q. DNA from the sporadic ovarian tumour did not show LOH in the region of BRCA1 but loss was confined to distal 17q.

To determine if this insertion caused a splice site mutation, we designed primers within exons 20 and 21 which amplified a 110 bp fragment by RTPCR in both control RNA and RNA from the archival tumour samples. Therefore it appears that this 12 bp duplication is a rare sequence variant, segregating with the disease.

### Expression of connexin 43 and connexin 26 in human breast tumours

*S Jamieson[1], JJ Going[2], R D'Arcy[1] and WD George[1]*

[1]Dept. of Surgery, Western Infirmary, Glasgow G11 6NT, [2]Dept. of Pathology, Royal Infirmary, Glasgow G4 0SF

It has been proposed that gap junctional intercellular communication (GJIC) plays a role in tumour suppression[1]. In support of this idea, Lee et al[2] found that the gap junction gene products connexin 43 (Cx43) and connexin 26 (Cx26) were present in normal human mammary epithelial cells but were lost in human breast tumour cell lines.

*© Cancer Research Campaign 1997*

We have investigated expression of Cx43 and Cx26 in normal human breast, 11 benign tumours and 27 invasive carcinomas of the breast. Tissues were snap-frozen in liquid nitrogen and 5 μm cryostat sections were acetone-fixed and processed for immunocytochemistry, using a biotin-streptavidin technique. Mouse monoclonal antibodies (Zymed) were raised against synthetic peptides corresponding to Cx43 and Cx26 sequences.

We confirm previous reports of intercellular punctate Cx43 expression in the myoepithelium of normal human breast[3,4]. We did not detect Cx26 in normal human breast, although we did obtain clear punctate staining in the sweat duct and hair follicle (outer root sheath) of control skin sections. Others have previously failed to detect Cx26 in human breast in vivo[3,5] and whilst Monaghan et al found Cx26 immunolabelling of ductal epithelial cells, they could find no ultrastructural evidence for GJs in these cells[4].

Cx43 expression in fibroadenomas and a benign phyllodes tumour resembled normal breast: only myoepithelial cells expressed Cx43 and there was no expression of Cx26. Some invasive carcinomas showed heterogeneous staining for Cx26 in the carcinoma cells, but this was always cytoplasmic. Over half of the invasive carcinomas showed heterogeneous staining for Cx43 in the carcinoma cells and occasionally this was punctate and intercellular. In addition, all carcinomas expressed punctate Cx43 on the plasma membrane of elongated cells within the stroma, most probably myofibroblasts.

Expression of Cx43 by myoepithelial cells and myofibroblasts is consistent with expression of this connexin in other contractile cells (cardiac muscle and myometrium). The aberrant expression of Cx43 and Cx26 in invasive carcinomas is not inconsistent with a role for GJIC in tumour suppression, but the heterogeneous and predominantly cytoplasmic nature of this expression may simply reflect the genetic and phenotypic instability of these tumours.

#### REFERENCES

1. Yamasaki H (1990) Immunol Ser 51: 245
2. Lee SW et al (1992) J. Cell Biol 118: 1213
3. Pozzi A et al (1995) Exp Cell Res 220: 212
4. Monaghan P et al (1996) Exp Cell Res 223: 29
5. Wilgenbus KK et al (1992) Int J Cancer 51: 522

### Apoptosis is induced but Bcl-2 is unaffected by tamoxifen and ICI 182780 in primary breast cancer

*M Dowsett[1,2], PA Ellis[1,2], S Johnston[1,2], R Clarke[3], E Anderson[3], A Howell[3], J Salter[1], S Detre[1], R Nicholson[4], J Robertson[5], IE Smith[2]*

[1]Depts of Academic Biochemistry, and the [2]Breast Unit, Royal Marsden Hospital, London; [3]Dept of Medicine, Christie Hospital, Manchester; [4]Tenovus Inst., Cardiff; [5]Dept Surgery, The City Hospital, Nottingham

Hormonal breast cancer therapies have traditionally been considered cytostatic but pre-clinical data indicate that anti-oestrogens can induce apoptosis, possibly by reversing oestrogen-induced upregulation of Bcl-2.

We have measured apoptosis, proliferation, and Bcl-2 expression in primary breast cancer (PBC) patients before and after presurgical treatment (Rx) with 20 mg/day Tamoxifen (TAM; study 1) or 6 or 18 mg/day of the pure anti-oestrogen ICI 182780 (ICI; study 2). In each study there was a randomised no-Rx (NT) control group. In study 1 median time to surgery was 21 days (range 6–65 days). In study 2 presurgical Rx was for 7 days in all patients. In study 1, apoptosis and proliferation were incorporated as a growth index (GI) for each patient.

Proliferation was reduced in both Rx groups but not in the NT groups (previously published). TAM significantly increased apoptotic index (AI) in ER+ve ($P < 0.05$) but not in ER–ve tumours. GI in ER+ve TAM-treated patients was significantly lower than in NT patients ($P = 0.01$) but there was no difference between the two groups in ER–ve patients. There was a significant increase in AI following Rx with ICI (median pre-Rx 0.34%, post-Rx 0.97%, $P = 0.003$). Insufficient pairs of samples were available to determine whether this change was confined to ER+ve tumours, but AI was significantly higher in excision biopsies from ICI-treated than from NT patients for ER+ve ($P = 0.005$) but not ER-ve tumours ($P = 0.16$). Bcl-2 was strongly associated with ER status before and after TAM ($P = 0.001$ and 0.005, respectively). There was a trend for Bcl-2 to be reduced in TAM-treated patients ($P = 0.06$), but this trend was not confirmed in ER+ve patients. Bcl-2 expression in excision biopsies was not significantly different between ICI-treated and NT patients overall or according to ER status. There was no significant correlation between AI and Bcl-2 in either pre- or post-treatment TAM or ICI study groups.

This study provides the first evidence in patients that apoptosis is induced in ER+ve primary breast cancer by both non-steroidal and steroidal anti-oestrogens. This does not appear to be due to changes in Bcl-2 expression.

### An unusual p53 germline mutation in the oligermerisation domain in a Li-Fraumeni-like family

*ME Lomax[1], DM Barnes[2], R Gilchrist[1], SM Picksley[3], JM Varley[4], RS Camplejohn[1]*

[1]Richard Dimbleby Dept. Cancer Res., UMDS, St. Thomas' Hosp., London SE1 7EH, [2]ICRF Clin. Oncol. Unit, Guy's Hosp., London SE1 9RT, [3]CRC Cell Transformation Group, Dept. Bioch., Univ. Dundee, Dundee DD1 4HN, [4]CRC Dept. Cancer Genetics, Paterson Inst. Cancer Res., Manchester M20 9BX

Investigations performed by Barnes et al (Lancet 340, 259, 1992) demonstrated that patient 904005 had elevated levels of p53 protein in both the malignant and non-malignant cells of a breast tumour. This unusual staining pattern was also seen in keratinocytes, fibroblasts and endothelial cells at a site distant from the tumour. The p53 protein could not be precipitated by the PAb240 antibody, which is specific for p53 protein in the mutant conformation, suggesting the p53 protein was wild-type in this patient. Quantitative ELISA tests and Western blotting confirmed that normal tissues from patient 904005 express higher levels of p53 protein when compared to cells from normal individuals. Despite extensive investigation, with the emphasis on the core DNA binding domain, no germline mutation was found in the p53 gene (Barnes et al, 1992). In the present study, two recently described functional assays for p53, an apoptotic assay and the yeast based FASAY, have been employed to study the functional activity of p53 in this patient. The results of the apoptotic assay

© Cancer Research Campaign 1997

suggest a p53-related defect and the results of the FASAY point strongly to this defect being an actual p53 gene mutation. The sequencing of the whole cDNA rescued from the yeast colonies (codons 67 to 347) revealed a CGC to TGC mutation at codon 337, producing an Arg to Cys substitution. Sequencing of exon 10 of genomic DNA confirmed there was an Arg to Cys substitution at codon 337. This is an unusual mutation as it is in the oligomerisation domain of the p53 gene, not the core DNA binding domain which is the usual site of p53 mutations. The site of the mutation in the p53 gene of this patient explains why the p53 protein could not be precipitated by the PAb240 antibody. In summary, it is possible for functional assays, such as the apoptotic assay and FASAY, to detect a wide spectrum of p53-related defects with minimum time and effort, properties which are important if large numbers of LFS/LFL family members are to be screened for p53 mutations.

### Msp1 polymorphism of the CYP4501A1 is not associated with increased risk of NSCLC in NW England

*HL Ross[1], T Liloglou[1], A Swift[1], JR Gosney[2], RJ Donnelly[3] and JK Field[1]*

[1]Molecular Genetics and Oncology Group,[2] Department of Pathology, University of Liverpool, L69 3BX. [3]Cardiothoracic Centre, Thomas Drive, Liverpool L14 3PE

In 1991 32 000 deaths in the UK were due to lung cancer. Most of these cases were smoking related. However, only a relatively small proportion of smokers develop lung cancer. It has been proposed that individual differences in xenobiotic metabolism could place a subset of the population at greater risk of developing lung cancer.

Genetic polymorphisms, particularly those in the cytochrome P450 superfamily, have been studied in a number of populations to see if they are over-represented in lung cancer patients. CYP4501A1 is expressed only in the lungs of smokers and has been shown to exhibit two polymorphisms. The first is the substitution of valine for isoleucine due to a change in exon 7 and the second is Msp1 cleavage.

We studied the prevalence of the Msp1 polymorphism in patients with non-small cell lung cancer (NSCLC) to determine if this was seen more frequently than would be expected in a control population and could therefore be used as a marker for high risk populations.

Restriction fragment length polymorphism (RFLP) was used to examine the 3' flanking region of CYP4501A1 from 58 NSCLC and 28 controls. Of 58 samples 13 were found to contain the Msp1 polymorphism, a frequency of 22.4%. This is not significantly different from the frequency in the control population at 21.4% (6/28). This would suggest that the Msp1 polymorphism in the CYP4501A1 gene is not associated with an increased risk of developing lung cancer. This finding is in contrast to a previous study on a Japanese population. It is possible that a combination of polymorphisms, not only in the P450 superfamily but in other enzymes involved in xenobiotic metabolism, may give a more accurate guide to elevated lung cancer risk.

This research was supported by the Roy Castle Cause For Hope Foundation.

### PCR/ligase chain reaction (PCR/LCR) and amplification refractory mutation system (ARMS) techniques to assess K-*ras* mutations for early lung cancer detection

*FM Scott[1], R Modali[2], TA Lehman[2], M Seddon[2], K Kelly[3], E Dempsey[4], V Wilson[5], M Tockman[6], G Ellison[7], J Fox[7], JK Field[1] and JL Mulshine[8]*

[1]Molecular Genetics and Oncology Gr., Clinical Dental Sciences, Univ. of Liverpool, L69 3BX, [2]BioServe Biotechnologies Ltd, 1050 West Street, Laurel, MD 20707, [3]Univ. of Colorado Health Sciences Center, Denver, CO 80262, [4]Dept. of Veterans Affairs Medical Ctr., Denver, CO 80262, [5]Louisiana State Univ., Baton Rouge, LA 70803, [6]Johns Hopkins Univ., Baltimore, MD 21205, [7]Zeneca Diagnostics, Gadbrook Park, Northwich, Cheshire CW9 7RA, and [8]NCI-Biomarkers and Prevention Research Br., Rockville, MD 20850

Mutations of the *ras* family of oncogenes are the most frequently observed genetic lesions in a number of human malignancies. About 80 to 90% of activating mutations of the K-*ras* gene occur at codon 12 or 13 in human non-small cell lung cancer. Methods are needed to screen rapidly for multiple mutations at K-*ras* codon 12 in premalignant clinical specimens such as bronchial washings. Two techniques have been developed for K-*ras* mutation detection: a polymerase chain reaction/ligase chain reaction (PCR/LCR) technique and ARMS. The PCR/LCR technique allows multiplex determination of K-*ras* mutations in the first base of codon 12. ARMS primers have been developed for determination of both first and second base K-*ras* codon 12 mutations. PCR/LCR has been applied to human bronchial lavage (BL) specimens and 84% of the BL specimens examined contain at least one mutation in K-*ras* codon 12. By comparison, 9% of lung tumours tested by ARMS displayed a mutation. The PCR/LCR and ARMS techniques have similar sensitivities. The high percentage of specimens with K-*ras* mutations detected by PCR/LCR may indicate the widespread presence of K-*ras* mutations on the bronchial epithelium as sampled by BL.

Portions of this work are funded by a generous grant from the G. Harold and Leila Y. Mathers Charitable Trust. Supported in part by SPORE number CA58187-01 in the Lung Cancer Tissue Laboratory at the University of Colorado Cancer Center and SPORE number CA58184-01 at Johns Hopkins University.

### The gastrin gene is activated early in the progression of colonic polyps and Berrett's oesophagus

*AM Smith[1], P Clarke[1], N Griifin[2], A Varro[3], JD Hardcastle[1] and SA Watson[1]*

[1]C.S.U., Dept. of Surgery, [2]Dept. of Pathology, University of Nottingham, [3]Dept. of Physiology, University of Liverpool

**Introduction:** The gastrin gene has been shown to be activated in colorectal and oesophageal adenocarcinoma. Both precursor and mature gastrin peptides have been identified in tumour cells. These peptides have been shown to have a proliferative effect and it has been postulated that they act via autocrine/paracrine pathways.

The aim of this study was to determine the point in the progression of colonic polyps and Barrett's oesophagus the gastrin gene becomes activated.

 *© Cancer Research Campaign 1997*

**Methods:** Samples were taken from patients with colorectal polyps ($n = 55$). Polyps ranged from tubular adenomas < 1 cm to polyp cancers ($n = 5$, per group). Biopsies were taken from patients with Barrett's oesophagus ($n = 30$, including all forms of metaplasia and types of dysplasia). Samples were also taken from normal colonic ($n = 5$) and oesophageal mucosa ($n = 5$). Paraffin embedded specimens were stained with polyclonal antisera against progastrin glycine-extended gastrin, amidated gastrin and the gastrin/CCKB receptor. Binding of the primary was detected using two techniques, the avidin-biotin-peroxidase and alkaline phosphatase red methods. Samples were assessed blind by two independent observers for site and degree of staining. Expression of the gastrin and gastrin/CCKB receptor gene was confirmed by use of the reverse transcriptase polymerase chain reaction.
**Results:** Immunocytochemistry demonstrated that progastrin, glycine-extended gastrin, amidated gastrin and the gastrin/CCKB receptor are expressed at high levels in the cytoplasm of epithelial cells in tubular adenomas < 1 cm and at a similar level in all forms of Barrett's metaplasia with no dysplasia. Gene products continue to be over-expressed through all stages of progression in the colon and oesophagus. Gastrin was not expressed in normal oesophageal mucosa. In normal colonic mucosa expression was confined to the neuroendocrine cells.
**Conclusions:** The gastrin gene is expressed at an early point in the progression of both colonic polyps and Barrett's oesophagus. It may have a proliferative effect as all components for an autocrine growth pathway are present. Expression of the gastrin gene early in the development of adenocarcinomas at two sites in the gastrointestinal tract may represent the reactivation of a dormant fetal growth factor.

### Determination of genetic events in oral cancer and precancer provides insight into the molecular pathogenesis of early disease and prognostic information

*M Partridge[1], G Emilion, S Pateromichelakis, R A'Hern[2], G Lee, E Phillips, M Falworth, JD Langdon*

[1]King's College Hospital and [2]Royal Marsden Hospital, London

Some oral cancers are preceded by potentially malignant lesions which show varying degrees of dysplasia. The clinical appearance of these lesions does not predict risk of transformation and markers are needed to identify high risk lesions. Once cancer develops, treatment depends on the extent of tumour spread and histological features. However, the 5-year survival rate for oral cancer is < 50%, suggesting that our current staging techniques and treatment protocols may not give our patients the best chance of cure.

We have used microsatellite assay with 51 markers to compare dysplastic lesions which progressed to cancer and a group of lesions which have not progressed, and screened primary SCC for allelic imbalance at loci likely to harbour tumour suppressors for this disease. The aim of the study was to help build databases of relevant events, to see whether profiling the spectrum of genetic abnormalities, in conjunction with studies determining gene expression at the protein level, can provide insight into the molecular pathogenesis of this disease as well as useful diagnostic and prognostic information.

The analysis reveals that some matched dysplastic and malignant lesions show genetic abnormalities in the dysplasia which are not present in the corresponding tumour. This suggests that these lesions arise from different clones and supports the concept of field change within the oral cavity. However, other matched lesions show similar genetic changes, suggesting that migration of altered epithelial cells also occurs. Allelic imbalance at chromosomal regions at 3p21, 3p13-12, 9p21, p53 and DCC was the most frequent early event in the malignant process. Deletion mapping has provided valuable insight into the chromosomal areas implicated in this disease. The number of loci showing abnormalities was increased in potentially malignant lesions which progressed, and with advancing tumour stage.

A statistically significant relationship was observed between the percentage of loci showing allelic imbalance (AI) and overall survival. Patients were divided into three approximately equally-sized groups on the basis of their percentage of AI. The relative death rates in the low, intermediate and high imbalance groups were 1.0, 2.47, and 6.10 respectively ($P < 0.001$; the group with low AI was used as the reference group). Multivariate analysis using Cox's regression indicated that this relationship was independent of stage. This suggests that alteration of multiple growth regulatory pathways in a given tumour may help identify aggressive lesions. Identification of a small number of key commonly occurring deletions and mutations within each primary cancer will also enable us to refine our existing staging techniques by providing the basis for molecular methods to screen for disseminated disease.

### Amplification and increased dosage of the telomerase RNA gene in human cancer

*AI Soder[1], SF Hoare[1], JJ Going[3], S Muir[1], EK Parkinson[2], WN Keith[1]*

[1]CRC Dept. Medical Oncology, University of Glasgow, and [2]Beatson Institute for Cancer Research, CRC Beatson Labs., Garscube Estate, Switchback Rd, Glasgow G61 1BD, UK. [3]Dept. of Pathology, Glasgow University

**Aim:** Telomerase activity has been linked to cellular immortality and tumour progression and has been discussed as a target for chemotherapy. The aim was therefore to investigate genetic alterations involving components of the telomerase enzyme complex that could lead to increased telomerase activity.
**Methods:** A genomic clone containing the human telomerase RNA gene was identified by screening the Du Pont Merck Pharmaceutical Company human foreskin fibroblast P1 phage library. Screening was carried out by Genome Systems Inc., St. Louis, Missouri, using PCR primers directed to the hTR genomic sequence. A clone was confirmed as containing hTR sequences by the sequencing of PCR products generated using clone as a template. This clone was mapped using fluorescence in situ hybridisation (FISH). Interphase cytogenetic analysis was carried out on frozen sections from 73 biopsies from cervix, lung and head and neck cancers using the cloned hTR sequences to quantitate hTR gene copy number.
**Results:** We have accurately mapped the human telomerase RNA gene to chromosome 3q26.3, a region frequently subject to copy number gains in human tumours. The hTR gene was amplified in 4 tumours, (2/33 cervix, 1/31 SCC-HN, 1/9 lung tumours), and the pattern of hybridisation indicated that the amplified sequences were present either as integrated blocks or extrachromosomally. In addition, increased copy numbers of the hTR locus was also observed with up to ten copies per nucleus, and hybridisations with

© Cancer Research Campaign 1997

a chromosome 3 centromeric probe indicated that polysomy and isochromosome 3q formation may contribute to this.

**Conclusions:** Our results are the first report of a genetic alteration involving a known component of telomerase in human cancer and support the notion that telomerase may be reactivated in some human tumour types. Indeed, it is also the first report of the amplification of a specific locus within the chromosome 3q region frequently subject to copy number gains in human tumours.

### The molecular pathogenesis of polycytheamia vera

*FA Asimakopoulos[1], MA Aldred[1], JGR Gilbert[1], AJ Bench[1], PH Hollings[2] and AR Green[1]*

[1]University of Cambridge, Dept. of Haematology, MRC Centre, Hills Road, Cambridge CB2 2QH, [2]Wellington Hospital, Wellington, New Zealand

Acquired deletions of chromosome 20q are recurrent abnormalities associated with myeloproliferative disorders, especially polycythaemia vera (PV). The association of these deletions with 'stem cell' disorders suggests that they may mark the site of one or more genes, loss or inactivation of which perturbs the regulation of normal haematopoietic progenitors. We have previously used quantitative Southern blotting and microsatellite PCR to characterise 20q deletions at the molecular level (Asimakopoulos et al, *Blood*, 84: 3086, 1994). These studies demonstrated telomeric and centromeric breakpoint heterogeneity and defined a common deleted region in PV of 16–21 cM. Several candidate genes such as the tyrosine kinases *SRC* and *HCK* and the transcription factor *RBL1* were found to map outside this region. However, this critical region did contain known genes with a role in growth regulation.

Molecular analysis of blast DNA from a patient with PV/AML has subsequently reduced the common deleted region to exclude the genes for the tyrosine phosphatase *PTPN1* and transcription factor *CEBPbeta*. A YAC contig covering this region of 12–18 cM has been constructed to provide a physical map prior to isolation and mutational analysis of further candidate genes. In addition, two approaches have been used to reduce the size of the common deleted region:

Firstly, microsatellite PCR analysis of granulocyte DNA from 48 patients with polycytheamia vera and no known 20q abnormalities demonstrated that the main mechanism for loss of heterozygosity of chromosome 20q is interstitial deletion.

Secondly, an increasingly large panel of samples from patients with 20q deletions has been analysed. Interestingly, none of the deletion breakpoints fell within the common deleted region. Taken together, these observations raise the possibility that simultaneous deletion of several genes on chromosome 20q may be required to produce a phenotypic effect.

### Will molecular techniques supplant immunocytochemistry in the detection of occult melanoma cells in regional lymph nodes?

*AJ Cochran[1,2], J Guo[1], RR Huang[1], D-R Wen[1]*

Departments of Pathology and Laboratory Medicine[1] and Surgery, Jonsson Comprehensive Cancer Center[2], UCLA School of Medicine, Box 951732, Los Angeles, CA, 90095–1713, USA

Immunohistochemistry (IHC) with antibodies to S-100 protein and HMB-45 detects occult melanoma cells in lymph nodes tumour-free on examination of HE stained sections. By this approach 14% of patients with high risk melanoma had occult melanoma in at least one node (Cochran AJ, Surg Path 12:612, 1988) and 29% of ostensibly melanoma-free nodes in lymphadenectomy specimens from patients with node spread melanoma contained tumour (Cochran, Int J Cancer 34:159, 1984). The sensitivity of this approach has been widely confirmed. IHC evaluation of 'sentinel' lymph nodes is the basis of the techniques of lymphatic mapping and selective lymph node dissection (Morton, Arch Surg 127:392, 1992), for high risk melanoma and other cancers. The approach requires identification of the sentinel node, the first node on the direct lymphatic drainage pathway from the primary and accurate determination as to whether that node contains tumour. Absent tumour, no further treatment is undertaken. If tumour is present a full lymphadenectomy is performed. When examined by solution phase PCR, using a primer for m-tyrosinase, some nodes that do not contain melanoma by IHC give a positive signal for m-tyrosinase. Does this certainly indicate the presence of occult tumour? There are several potential sources of m-tyrosinase in nodes that could provide a confounding signal, including nerve-associated Schwann cells and nevocytes in the node capsule (Carson, Surg Path 20:834, 1996). The true source of signal can be determined only by techniques, such as in situ RT-PCR, that combine cytologic identification of the source of signal with amplification of signal from single cells to readily detectable levels. We will demonstrate the successful application of in situ RT-PCR to melanoma cells in culture and in sections from formalin-fixed and paraffin embedded blocks. Application of these in situ approaches to the sentinel nodes will disclose whether all nodes that contain m-tyrosinase on solution phase PCR actually contain occult melanoma. (NCI CA2960)

### Detection of neuroblastoma cells by RT-PCR for tyrosine hydroxylase

*SA Burchill[1], RA Harrison[1], FM Bradbury, P Selby[2] and IJ Lewis[3]*

[1]Children's Cancer Research Laboratory, [2]Cancer Medicine Research Unit and [3]Paediatric Oncology, SJUH, Leeds LS9 7TF

Disseminating disease is important in the clinical outcome for patients with neuroblastoma. We have examined detection of neuroblastoma cells in peripheral blood (PB), bone marrow (BM) and peripheral blood stem cell harvests (PBSCH) by RT-PCR for tyrosine hydroxylase (TH).

Using this method it is possible to detect a single neuroblastoma cell in 1 ml of whole blood or bone marrow. No TH mRNA was detected in control PB (20), BM (6) or PBSCHs (4). TH mRNA was detected by RT-PCR in all 16 neuroblastomas examined.

A strong correlation was found between detection of TH mRNA by RT-PCR and metastatic disease status detected by conventional methods in 40 patients. In two patients, neuroblastoma cells detected by RT-PCR for TH mRNA at diagnosis were detected throughout and at the end of therapy. These patients appeared not to respond to therapy and relapsed within 12 months of diagnosis. Both these patients have subsequently died of their disease.

Analysis of blood samples from 30 patients identified circulating tumour cells in 26% clinically disease free patients. On follow-up 71% of patients with circulating tumour cells relapsed and died, compared to 10% of those without. TH mRNA transcripts were

*© Cancer Research Campaign 1997*

detected in peripheral blood up to 12 months before urinary catecholamine levels began to increase and clinical relapse occurred. Comparative analysis of blood samples before clinical relapse and at the time of relapse demonstrated an increase in circulating TH mRNA at relapse, suggesting an increase in the number of circulating tumour cells.

In summary, RT-PCR for TH is a sensitive and specific method for the detection of circulating disease in neuroblastoma. This method may be used to monitor BM or PBSCHs for contaminating tumour cells, though the precise risk associated with tumour cell contamination of BM or PBSCHs used in engraftment is not clear. Preliminary evidence demonstrates the level of contamination may be substantial and will vary depending on the time of harvest. Longterm clinical significance of neuroblastoma cell detection is currently being evaluated through the UKCCSG study number NB 9305.

Presented on behalf of the United Kingdom Children's Cancer Study Group.

### Characterisation of small round cell tumours of childhood by tissue specific RT-PCR

*SA Burchill[1], C Cullinane[2], J Wheeldon[1], P Roberts[3] and IJ Lewis[4]*

[1]Cancer Research Unit, [2]Depts of Pathology, [3]Cytogenetics and [4]Paediatric Oncology, SJUH, Leeds LS9 7TF

Small round cell tumours in children are often difficult to distinguish from each other using conventional methodologies. The aim of this study was to identify potential targets for RT-PCR characterisation of small round cell tumours and compare this with standard histopathology and cytogenetics. Targets chosen for study included tyrosine hydroxylase (TH) for neuroblastoma, MyoD1 for rhabdomyosarcomas, EWS-FLI1 fusion transcripts for Ewing's and pPNETs, and the PAX3-FKHR fusion transcript for alveolar rhabdomyosarcomas.

RNA was extracted from small round cell tumours including neuroblastoma, pPNET, Ewing's and rhabdomyosarcoma. This RNA was reverse transcribed to produce cDNA and using polymerase chain reaction amplified for expression of TH, EWS-FLI1, PAX3-FKHR and MyoD1 mRNA. Extraction of RNA, subsequent reverse transcriptase polymerase chain reactions and analysis of products was performed in designated lamina flowhoods and rooms. The identity of the amplified products was confirmed by liquid hybridisation and sequence analysis.

Good quality RNA was extracted from all tumour samples; all were positive for the house keeping gene β2-microglobulin. 11/11 neuroblastomas were positive for TH, 6/6 rhabdomyosarcomas were positive for MyoD1 and 6/6 Ewing's/pPNETs positive for EWS-Fli1 mRNA, demonstrating a strong correlation between RT-PCR and conventional analysis. However, 30% of these tumours were bi or triphenotypic. 2 neuroblastomas and 1 rhabdomyosarcoma were positive for EWS-FLI1 mRNA, and 1 Ewing's was also positive for TH. Two rhabdomyosarcomas and 1 pPNET expressed mRNA for EWS-FLI1, MyoD1 and TH. None of the samples expressed mRNA for the PAX3-FKHR translocation.

In summary, analysis by RT-PCR for tissue specific gene expression appears to aid differential diagnosis of small round cell tumours. Although these results demonstrate expression of the chimeric EWS-FLI1, TH or MyoD1 transcripts in phenotypically distinct tumours, a subset of the tumours examined demonstrate characteristics of mixed phenotype. The presence of EWS-FLI1 mRNA in neuroblastomas and rhabdomyosarcomas suggests EWS-FLI1 transcripts are not necessarily pathognomonic of Ewing's and pPNETs. These results may have implications for understanding small round cell tumour biology.

### Identification of a protein phosphatase 2A regulatory subunit differentially expressed in malignant cells

*GF Francia, R Poulsom[1], SD Mitchell, JF Marshall and IR Hart*

Richard Dimbleby Dept./ICRF Unit, Rayne Institute, St Thomas' Hospital, London SE1 7EH. [1]ICRF Histopathology Unit, Lincoln's Inn Fields, London WC2A 3PX

We previously reported the identification by rtPCR of four transcripts differentially expressed between murine B16F10 (metastatic melanoma) and Melan-a (immortalised melanocyte) syngeneic cell lines (Francia et al, *Cancer Res* 56: 3855 1996). We now show that one such transcript is the mouse homologue of the human B56γ regulatory subunit of protein phosphatase 2A(PP2A). By Northern blotting analysis this transcript is detected only in the metastatic B16F10 cells but not in Melan-a nor in simply tumourigenic (Melan-ab/LTRras) or weakly metastatic variants (B16F1). The full coding region of the murine homologue has been cloned and found to be 90% identical to its human homologue. Using homologous probes we have examined expression of the human gene in neoplastic tissue by two procedures, Northern blotting and in situ hybridisation.

Northern blotting analysis of the human B56γ regulatory subunit showed overexpression in human melanoma cell lines established from metastatic disease (A375P, A375M, HMB2) relative to normal epidermal melanocytes. By in situ hybridisation a weak tissue-wide distribution was observed with evidence of overexpression in carcinomas of the ovary and colon. These data show that a murine gene, detected as being differentially expressed in a model of tumour progression, has a human counterpart which shows comparable variation of activity. Since PP2A activity is regulated by B56γ it seems possible that this association reflects a difference in phosphatase activity in malignant tissue.

### Prevalence and prognostic significance of expression of p53 and bc1–2 gene proteins in Ewing's sarcoma of bone

*A Abudu, DC Mangham, GM Reynolds, PB Pynsent, RM Tillman, SR Carter and RJ Grimer*

Royal Orthopaedic Hospital Oncology Service, The Royal Orthopaedic Hospital, Birmingham B31 2AP, UK

Immunohistochemistry was used to study abnormalities of p53 and bc1–2 genes in biopsy tissues of 52 patients with Ewing's sarcoma of bone to determine their significance in diagnosis and prognosis of the disease. Mean age was 17 years. Minimum

© Cancer Research Campaign 1997

follow-up was 30 months. Tumours were located in the extremities and central bones in 35 and 17 patients respectively. Seven patients had metastases at diagnosis.

p53 protein was expressed in 7 patients (14%) and bcl–2 in 12 patients (23%). There was no relationship between expression of these proteins and tumour stage, site and necrosis following chemotherapy ($P > 0.5$). Five year relapse-free survival and overall survival in patients without metastases at diagnosis were 66% and 71% respectively in p53 protein negative patients compared to 20% relapse-free and overall survival in those with p53 protein expression ($P = 0.01$). The poorer prognosis in p53 protein positive patients was independent of site, local treatment or necrosis of the tumours ($P = 0.02$).

bcl–2 had no influence on survival. There was no statistical relationship between expression of bcl–2 and p53 proteins. Three patients expressed both bcl–2 and p53 proteins but the disease course in these patients was not different from the course of disease in patients with expression of just one protein.

p53 protein is an independent poor prognostic factor in Ewing's sarcoma of bone. bcl–2 protein expression has no prognostic value or relationship to expression of p53 proteins in patients with Ewing's sarcoma.

### Molecular cloning and expression of the human hyaluronan receptor rhamm in breast cancer cells

*V Assmann, JF Marshall and IR Hart*

R Dimbleby Department of Cancer Research/ICRF Laboratory, The Rayne Institute, St Thomas' Hospital, London SE1 7EH

A characteristic of invasive, as compared with in situ breast carcinoma cells, is the acquisition of motility enabling them to penetrate the epithelial basement membrane and enter the underlying ECM. We have investigated the potential role of the hyaluronan (HA) receptor RHAMM (receptor for hyaluronan-mediated motility) in breast cancer. RHAMM has been shown to mediate the HA-dependent migration of various cell types including ras-transformed fibroblasts, B- and T-lymphocytes. We have isolated a cDNA clone which codes for the human HA-receptor RHAMM by screening of a lambda gt11 expression library, derived from the endometrium carcinoma cell line Ishikawa, using PCR-based approaches. The predicted human RHAMM sequence possesses significant homology to the murine RHAMM sequence (84% homology in the overlapping region). A single RHAMM gene transcript is expressed in the eight breast cancer cell lines investigated by RT-PCR analysis and Northern blot hybridisation. To examine the expression of the RHAMM protein we have raised a polyconal antibody against a bacterially expressed glutathione-S-transferase (GST)-RHAMM fusion protein. The antiserum detects an 85–90 kDa protein in Western blot analysis and is expressed by all the breast cancer cell lines examined. Surprisingly, the anti-RHAMM antibody does not label the cell surface, but the cytoplasm and nucleus, of breast cancer cells in immunofluorescence studies. In addition, RHAMM proteins were not accessible to surface labelling by iodination or biotinylation. In contrast, the 85 kDa RHAMM protein was immunoprecipitated from 35S-methionine-labelled whole-cell extracts indicating an intracellular localisation of the antigen. Preliminary data from immunohistochemical staining of primary breast cancers showed an increased expression of RHAMM in the cytoplasm and the nucleus of tumour cells compared to normal breast epithelium. In addition, RHAMM gene transcripts were barely detectable in normal breast epithelial cells by Northern blot analysis. Taken together, our findings indicate the RHAMM is likely not to function as a cell motility receptor in human breast cancer cells but instead its intracellular localisation strongly suggests that RHAMM belongs to a novel family of intracellular hyaluronan-binding proteins.

### Stimulation of pro-MMP-2 release in cultures of human fibroblasts and ovarian carcinoma cells

*RS Boyd and FR Balkwill*

Biological Therapies Laboratory, ICRF, London WC2A 3PX, UK

Ovarian carcinoma contains elevated levels of MMP-2, much of which is in an activated form. Co-culture of human fibroblasts, dermal and breast tumour derived, and the ovarian carcinoma cells, PEO1, PEO14 and SKOV-3, resulted in an increase (2–8-fold) in supernatant pro-MMP-2 levels, as determined by quantitative gelatin zymography. This interaction between fibroblasts and carcinoma cells was found to involve both soluble factor(s) and cell–cell contact. Cell contact was the most important component of the interaction. Use of RT-PCR indicated that both fibroblasts and carcinoma cells expressed mRNA for EMMPRIN, a recently described membrane bound stimulator of pro-MMP-2 release. PE014, SKOV-3 and fibroblast cells were found to express mRNA for the pro-MMP-2 activator, MT-MMP 1, and MT-MMP 1 protein could be detected in the human dermal fibroblasts. Co-culture of carcinoma and fibroblast cells, however, resulted in only a minor activation of supernatant pro-MMP-2 (< 5%). In conclusion, co-culture of ovarian carcinoma cells and fibroblasts results in an enhanced release of pro-MMP-2 but no activation of this enzyme.

### FGF1, FGF2 and FGF8 co-expression in human prostate cancer

*HY Leung[1], T Dorkin[1], C Marsh[2], M Robinson[2] and DE Neal[1]*

[1]Dept of Surgery, The Medical School, University of Newcastle NE2 4HH; [2]Dept of Pathology, Freeman Hospital, Newcastle NE7 7DN

**Introduction:** Fibroblast Growth Factors (FGFs) are important mitogens and may contribute towards to the pathobiology of human prostate cancer. We have recently described over-expression of FGF8, or androgen-induced growth factor, in prostate cancer (*Oncogene* 1996, 12, 1833). As different FGFs may interact with individual high affinity FGF receptors (FGFRs), we have now extended our study to examine the expression of FGF1 (aFGF) and FGF2 (bFGF) on the same cohort of cases.
**Method:** Thirty-one cases of prostate cancer (5 well differentiated, 10 moderately differentiated and 16 poorly differentiated) and 5 cases of benign prostatic hyperplasia (BPH) were used. Immunohistochemical analysis using specific antibodies against

 © Cancer Research Campaign 1997

human FGF1 and FGF2 were performed as described previously (*Cancer Res* 1993, 53, 4741). Negative control was performed for each case by omitting the primary antibody. Immunoreactivities were assessed without clinico-pathological details and were graded as negative, weak, moderate and strong.

**Results:** Over-expression of FGF1 and FGF2 were noted in the malignant prostatic epithelium. FGF1 expression was observed in 80% (25/31) of prostate cancer. High grade tumours appeared to express FGF1 at higher levels, but this assocition failed to reach statistical significance. FGF2 immunoreactivity was observed in 55% of prostate cancer; with no relation to tumour grade. Immunoreactivities for FGF1 and FGF2 were cytoplasmic and homogeneous among tumour cells. At areas of prostatic intraductal neoplasia and peri-neural invasion, high levels of FGF1 expression were observed. In the adjacent benign prostatic epithelium, the epithelial cells stained negative for FGF1 and FGF2. Staining for FGF1 and FGF2 within the stroma was only seen in small proportion of the cases and the signals were at low intensities. BPH did not show any significant staining for FGF1 and FGF2. FGF8 over-expression was previously found in 71% (22/31) of the prostate cancer in this series.

**Conclusions:** FGF1, FGF2 and FGF8 co-expression exists in human prostate cancer, with FGF1 being most commonly expressed, followed by FGF8 and FGF2. Their over-expression appeared to be restricted to the malignant prostatic epithelium. Future work on the expression and interaction between multiples of FGFs and their high affinity FGFRs will allow better understanding of the role of FGFs in prostate cancer.

### Transcription of the SCL gene in erythroid and CD34 positive primitive myeloid cells is controlled by a complex network of lineage-restricted chromatin-dependent and chromatin-independent regulatory elements

*B Göttgens[1], F McLaughlin[1], E-O Bockamp[1], AG Elefanty[2] and AR Green[1]*

[1]University of Cambridge, Dept. of Haematology, MRC Centre, Hills Road, Cambridge CB2 2QH, [2]The Walter and Eliza Hall Institute of Medical Research, Melbourne, Victoria, 3050, Australia

The SCL gene (also known as TAL-1) encodes a basic helix-loop-helix transcription factor that is essential for the development of all haematopoietic lineages. Rearrangements of the SCL gene are also the commonest molecular pathology associated with T cell acute lymphoblastic leukaemia. SCL is normally expressed in pluripotent haematopoietic stem cells and its expression is maintained during differentiation along erythroid, mast and megakaryocytic lineages, but is extinguished following commitment to other cell types. The mechanisms responsible for this pattern of expression are poorly understood, but are likely to illuminate the molecular basis for stem cell development and lineage commitment.

Here we describe the identification of 7 lineage-restricted DNase I hypersensitive sites in a 45 kb region spanning the murine SCL locus. Committed erythroid cells and CD34 positive primitive myeloid cells exhibited different patterns of DNase I hypersensitive sites. The function of each hypersensitive site has been studied using both transient and stable reporter assays in erythroid, primitive myeloid and T cells. Multiple cell-specific regulatory elements have been identified. In particular a 3′ element (+17HS/+18HS) functioned as a potent erythroid enhancer, an intragenic element (+7HS) functioned as a chromatin-dependent silencer in primitive myeloid cells and another intragenic element (+3HS) functioned as a chromatin-dependent enhancer in both erythroid and primitive myeloid cells. Our results represent the first description of several key components of a complex network of regulatory elements controlling SCL expression during haematopoiesis.

### Therapeutic potential of anti-EGFR antibodies in antagonising the effects of the EGF family of ligands

*H Modjtahedi[1], BD Cohen[2] and C Dean[1]*

[1]The Institute of Cancer Research, McElwain Labs, 15 Cotswold Road, Belmont, Surrey, UK, [2]Bristol-Myers Squibb Pharmaceutical Research Institute, 3005 First Avenue, Seattle, USA

Overexpression of the epidermal growth factor receptor (EGFR) accompanied by production of one or more of its ligands has been reported in a wide range of human malignancies and this in turn has been associated with a poorer survival in many of these patients. Since the ligand-induced activation of such cells occurs via receptors on the cell surface rather than intracellularly, such a system may form a suitable target for monoclonal antibody (mAb)-directed therapy. We have described previously the production and characterization of a number of mAbs which are directed against five distinct epitopes on the external domain of the human EGFR and have been investigating their potential for diagnostic and therapeutic application in oncology. We have shown recently that mAbs which block the binding of the EGF, TGFα, heparin binding EGF-related growth factor (HB-EGF) and betacellulin (BTC) to the EGFR also 1) block the ligand-induced tyrosine phosphorylation of the EGF receptor, 2) inhibit the growth both in culture and in vivo of tumours which overexpress the EGF receptor, and 3) can be administered safely and localise efficiently to metastatic lesions in patients with head and neck or lung cancer.

If these antibodies are to be therapeutically effective, they must also block the activation of the EGFR induced by any member of the EGF family of ligands. In the present study, we have investigated whether, like other EGFR ligands, amphiregulin (AR) transmits its biological effects following binding to the EGF receptor and whether such effects could be antagonised in the presence of anti-EGFR mAbs. We show that like the other EGFR ligands, AR could inhibit the growth in culture of EGFR overexpressing tumour cell lines, namely HN5, HSC-1 and MDA-MB468 cells and induce tyrosine phosphorylation of the 170 kDa EGF receptor on HN5 cells. These effects were blocked in the presence of mAbs ICR62 or ICR80. However, unlike the other ligands, AR was unable to A) prevent the binding of $^{125}$I-EGF or the mAbs $^{125}$I-ICR62 and $^{125}$I-ICR80 to EJ cells or B) enhance the binding of another anti-EGFR mAb ICR9 to the EGF receptor on EJ cells. These results indicate that, despite activating the EGF receptor, AR, unlike the other ligands, does not bind to this receptor. On the basis of our data we conclude that the AR-induced activation of the EGFR, which may be mediated via hetero-dimerisation and transphosphorylation by another receptor, can be blocked in the presence of anti-EGFR mAbs. (Supported by the Medical Research Council, UK.)

© Cancer Research Campaign 1997

### The role of TGF$\beta_1$ in human colon cancer

*JM Bowman[1], Z Taylor[1], MO Smith[1], JA Royds[2], J Lawry[1]*

Institute for Cancer Studies[1] and Department of Pathology[2], University Medical School, Beech Hill Road, Sheffield S10 2RX

TGF$\beta_1$ is one member of a family of regulatory proteins having variable effects depending upon the cell type, stage of differentiation and ambient growth conditions. It is generally considered to be growth inhibitory on cells of epithelial origin, and acts via three surface receptors (Type I, II and III) in regulating G1 to S-phase progression through the actions of the cyclin dependent kinase inhibitors (CDKIs) p15, p21 and p27. In colon tumour progression TGF$\beta_1$ switches to being growth stimulatory, and has been linked with recurrence and metastases.

In vitro proliferation studies were undertaken using flow cytometry to measure cell cycle and BrDu incorporation in three colon cell lines. TGF$\beta_1$ was growth inhibitory for HT29 cells and moderately so for HCT116 cells. In contrast, the SW742 line was bi-responsive, being growth inhibited by TGF$\beta_1$ in the presence of serum, and stimulated in the absence of serum. All cell lines expressed high levels of Type II receptor (low levels of Type I); both being required to generate a heterodimeric complex capable of signal transduction. Expression of both receptors was always down regulated by TGF$\beta_1$, which also modulated cell cycle related proteins. In the growth inhibited HT29 cell line p27 (Kip1) expression was significantly increased, which acts as an inhibitor of the cyclin E/CDK2 complex normally required for entry into the cell cycle; whilst p15 and p21 expression was reduced. In contrast, under growth stimulatory conditions, the SW742 line had low level p27, high levels of p15 and p21.

In vivo studies of 36 archival samples of colon adenocarcinoma and 10 adenomatous polyps, using immunohistochemistry, were carried out in parallel to determine the potential correlation between TGF$\beta_1$ synthesis, the expression of receptors Type I and II, CDKI and cyclins, and stage of the disease. In particular, it was found that Dukes' B samples were Type II receptor positive and Type I receptor negative, with the reverse situation in Dukes' C and D samples. This effect may result in a loss of response to the inhibitory effects of TGF$\beta_1$. Type I receptor may prove to be a prognostic marker of disease progression.

We believe this study represents the first link between TGF$\beta_1$, p27 expression and the control of G1 to S-phase progression via the cyclin E complex, with reciprocal effects detected in the expression of p15 and p21.

This research is funded by the Y.C.R.C.

### Recombinant mycobacteria as cancer vaccines

*AM Jackson[1], MA Chambers[2], X Zhu[1], J Haley[3], P Patel[1], RE Banks[1], MA O'Donnell[4], K James[3] and P Selby[1]*

[1]ICRF Cancer Medicine Research Unit, University of Leeds, [2]Molecular Genetics, Central Vetinary Labs., Weybridge, [3]Dept. Surgery, University of Edinburgh, [4]Dept. Urology, Beth Israel Hospital, Boston, USA

Intravesical immunotherapy with live BCG vaccine yields complete responses in 75% of patients with *CIS* of the bladder. Favourable responses are associated with elevated levels of urinary cytokines and observations have been made showing particularly low levels of several interleukins in patients with persistent malignancy. Although the mechanisms of action of BCG therapy are not fully understood, it is likely that the success of BCG is attributable to local infiltration of activated leukocytes and their specific and non-specific cytotoxic activities. We are investigating the feasibility of engineering cytokine-secretion by mycobacteria for cancer therapy and vaccination.

Recently *E. coli*-mycobacterial 'shuttle' vectors have been developed which use *hsp70* promoters to drive expression of a recombinant gene. Modified forms of these vectors include the BCG alpha-antigen signal sequence (which facilitates secretion of proteins) in fusion with the recombinant gene. An interesting feature of this expression system is the incorporation of a second *hsp60* promotor driving expression of *A. victoria* green-fluorescent-protein (*gfp*). When excited by UV or laser radiation *gfp* emits visible green light, making it particularly useful for localising and tracking mycobacteria. Using overlap-extension RT-PCR we cloned a number of cytokines from human and murine mRNA into 2 vectors, pMOD-8 and pMOD-12. Preliminary experiments were undertaken with *M. smegmatis* due to its non-pathogenic nature and relatively rapid growth in culture. The levels of cytokine expression as measured by specific ELISA were as follows; IL-7 (10 pg/ml), IL-8 (50 ng/ml), IL-15 (300 pg/ml), RANTES (20 ng/ml), MCP-1 (150 ng/ml).

The unique difficulties of recombinant gene expression in mycobacteria, our initial results on the biological activities of mycobacterial-derived cytokines, and the future uses for this system to express tumour antigens in conjunction with cytokines will be discussed.

### Characterisation of nitroreductase as a 'suicide gene' for cancer gene therapy: hypoxia and cell cycle effects

*SM Bailey*, R Gilchrist, RS Camplejohn and IR Hart*

Richard Dimbleby Dept. of Cancer Research, St Thomas' Hospital, London SE1 7EH

*E. coli* nitroreductase is a 'suicide gene' proposed for use in cancer gene therapy. We have shown previously that V79 Chinese hamster lung cells transduced with the nitroreductase gene exhibit increased sensitivity to the nitro compounds CB 1954 and nitrofurazone relative to control cell lines. We have now extended the study and characterised this 'suicide gene' system in terms of the mechanism of cytotoxicity and effect of hypoxia. MCF-7 human breast cancer cells were transfected with plasmids containing either the nitroreductase gene in the sense or antisense orientation, driven by the CMV promoter, or control vectors. Stable clones were selected with puromycin. Nitroreductase expressing clones were up to 525-fold more sensitive to CB 1954 and 75-fold more sensitive to nitrofurazone compared with the control cells. The existence of a large differential was confirmed by clonogenic assay. Marked cell cycle perturbations were induced in a V79 nitroreductase expressing clone (F83-2) following treatment with cytotoxic (~99% cell kill) concentrations of CB 1954 (0.01 mM), including slowing in progression of cells through the cycle followed by accumulation in the G2/M phase. This drug concentration caused no effect in a clone transfected with the antisense vector (F80-18) but cell cycle perturbations were observed when an equitoxic dose of CB 1954 (10 mM) was used. Similar results were obtained for MCF-7 cells.

 © *Cancer Research Campaign 1997*

Nitrofurazone also induced cell cycle perturbations in V79 cells but, interestingly, this compound appeared to completely block progression through the cell cycle, thus indicating a different mechanism of action to CB 1954. The effect of hypoxia on CB 1954- or nitrofurazone-induced cytotoxicity also was investigated. For the V79 nitroreductase expressing clone, F83-2, there was no difference between the sensitivity of cells to either drug under hypoxic versus aerobic conditions. In contrast, the V79 antisense clone (F80-18) was ~12-fold more sensitive to CB 1954 and 7-fold more sensitive to nitrofurazone under hypoxic compared with aerobic conditions. These data suggest that agents with a different mechanism of action may be useful for nitroreductase gene therapy. In addition to targeting nitroreductase-expressing cells, these compounds could operate as dual function agents since they are more cytotoxic to hypoxic tumour cells than to oxic counterparts.

### In vivo quantification of thymidine salvage as a pharmacodynamic endpoint of thymidylate synthase inhibition

*P Wells, RN Gunn, JC Matthews, A Hughes[2], G Taylor[2], I Raff[2], DR Newell[2] and P Price[1]*

[1]MRC Cyclotron Unit, Hammersmith Hospital, London W12 0HS, [2]Cancer Research Unit, Medical School, University of Newcastle upon Tyne, Newcastle NE2 4HH

3,4-Dihydro-2-amino-6-methyl-4-oxo-5-(4-pyridylthio)-quinazolone dihydrochloride (AG337) is a nonclassical thymidylate synthase inhibitor (TS) developed to overcome potential resistance mechanisms which limit activity of classical antifolates and is being evaluated in a Cancer Research Campaign (CRC) Phase I study. It is anticipated that thymidine salvage pathways will be activated in tissues following treatment. Positron Emission Tomography (PET) with [2-$^{11}$C]thymidine has been used to demonstrate this effect which may reflect the degree of TS inhibition.

Five patients with metastatic colorectal cancer were studied. [2-$^{11}$C]thymidine PET scans were performed on two separate occasions, one before treatment and one on the second day of therapy, one hour after oral administration of AG337 (250 mg/m$^2$). The thymidine scans were acquired over 60 minutes following intravenous administration of tracer doses of radiolabelled [2-$^{11}$C]thymidine (maximum of 555 MBq). Three paired control studies were performed at one week intervals with no intervening treatment to assess reproducibility. In addition to the tomographic data, arterial blood samples were acquired from which a plasma thymidine input function was derived. Spectral analysis is a deconvolution method used to calculate the tissue impulse response function (IRF) which was used to characterise uptake (IRF at 60 minutes) and delivery (initial IRF accounting for blood volume). The PET derived measure is expressed as the fractional retention of tracer at 60 minutes (FRT) which equals uptake/delivery, as previous work has suggested that correction for tracer delivery is necessary.

Data on five treated patients are described. For tumour there was a significant increase in FRT and uptake post AG337 treatment relative to control studies (paired test $P = 0.04$). A notable (but not significant) increase was seen for spleen, whereas no change was noted in liver regions sampled. These results are consistent with enhancement of the thymidine salvage pathway following TS inhibition, reflected by an increase in labelled thymidine concentration in tumours relative to normal tissue.

This work is supported by CRC Grant No SP2 193/0102 and Schering Plough.

### In vivo demonstration of $^{11}$C-Temozolomide uptake by human recurrent high grade astrocytomas

*CS Brock[1], JC Matthews[1], G Brown[1], ES Newlands[2] and P Price[1]*

MRC Cyclotron Unit, Hammersmith Hospital[1], and Dept. of Medical Oncology, Charing Cross Hospital[2], London

The oral prodrug temozolomide is an imidazotetrazine derivative with cytotoxic activity in brain tumours.

Four patients with recurrent high grade gliomas receiving temozolomide as part of the CRC Phase I & II trials were studied. Tracer doses (50 mcg) of temozolomide radiolabelled with $^{11}$C (555 MBq) in the 3N-methyl position were injected intravenously and repeat studies were performed during therapeutic treatment. This was preceded by a 60 second infusion of $H_2{}^{15}O$. Positron emission tomography (PET) scans were performed on a 953B Siemens NeuroPET scanner for 90 minutes ($^{11}$C-Temozolomide ($^{11}$C-Tem)) and 4–6 minutes ($H_2{}^{15}O$). Blood radioactivity was monitored for the scan duration using continuous arterial sampling.

Tumour and normal tissue regions of interest were defined and applied to the dynamic PET images producing tissue and tumour time activity curves (TACs) of the mean pixel radioactivity. Once corrected for radiolabel decay, body surface area and injected dose of tracer the areas under the curve (AUC) were calculated. Tissue and tumour perfusion were calculated from the measured $^{15}$O concentration in arterial blood and the PET $H_2{}^{15}O$ time activity curves by the numerical fitting of a Kety-Schmidt model.

There was no significant correlation detected between tumour perfusion and tumour ($^{11}$C-Tem) AUC ($r^2 = 0.2074$). It may be that changes in perfusion do not affect temozolomide uptake or a more confounding variable, such as blood–brain barrier disruption, have a greater effect on drug uptake as measured with AUC. There was no significant difference between tumour perfusion in the tracer-only and temozolomide studies during treatment, suggesting that there is no evidence that temozolomide alters perfusion. There was significantly greater uptake of $^{11}$C-Tem by tumour as compared with normal contralateral brain ($P = 7.7405e-6$, paired *t*-test). For AUCs corrected for perfusion there was a greater percentage difference between tumour and contralateral brain (uncorrected 66 ± 16%; corrected 134 ± 33%).

PET allows in vivo demonstration of normal tissue and tumour perfusion and drug pharmacokinetics.

Temozolomide was developed by the CRC and is now licensed to Schering Plough. This work is supported by CRC grant no SP2 193/0102.

### Universal immuno-PCR for ultrasensitive detection of DNA repair components and other antigens

*Marian Case[1], Alastair D Burt[2], Margaret F Bassendine[1] and Glenn N Major[1]*

Departments of Medicine[1] and Pathology[2], The Medical School, University of Newcastle, Newcastle upon Tyne NE2 4HH

© Cancer Research Campaign 1997

Incidental human exposure to potentially carcinogenic alkylating agents is likely to yield very low levels of tissue DNA damage (adducts). Detection and quantitation of such low-level damage is technically difficult. This is the case for the powerfully promutagenic DNA base lesion $O^6$-methylguanine, which may be produced in human DNA following exposure to methylating agents such as the environmentally ubiquitous nitrosamines. We have assessed the feasibility of using immuno-PCR, a potentially extremely sensitive antigen detection method that utilises polymerase chain reaction (PCR) from a secondary antibody-target DNA conjugate to detect the specific binding of a primary antibody to its antigen. The universality of a single protocol for immuno-PCR has not yet been confirmed. To examine this and assess the utility of immuno-PCR for detection of very low levels of DNA adducts in human tissues, we established the method using purified rabbit polyclonal antibodies recognising four biochemically diverse antigens: pyruvate dehydrogenase complex (PDC; oligomeric; $M_r$ 8.5 × $10^6$), the DNA repair enzyme $O^6$-methylguanine-DNA methyltransferase (MGMT; monomeric; $M_r$ 21 700), an oligopeptide (20-mer; $M_r$ 2310) and a DNA adduct conjugated to BSA ($O^6$-methylguanosine-BSA; $M_r$ ~ 67 600). Antigen detection limits by immuno-PCR were: ~ 3 × $10^{-4}$ amol or 180 molecules of $O^6$-methylguanosine, ~ 6 × $10^{-4}$ amol or 350 molecules of PDC, ~ 0.2 amol or 1.3 × $10^5$ molecules of oligopeptide, and ~ 115 amol or 700 × $10^5$ molecules of MGMT. Antigen instability was observed with MGMT in highly dilute solution, which may account in part for the lower detection limit. Results of this study indicate that (i) immuno-PCR is able to be used for the ultrasensitive detection of a diverse variety of antigens, (ii) increases in immuno-PCR detection limit are reflected by increases in primary antibody titres determined by ELISA, and (iii) the sensitivity of detection using immuno-PCR is independent of antigen $M_r$. We have yet to investigate the suitability of the method for detecting anything other than purified antigens. Nevertheless, our findings suggest that immuno-PCR may be of use for the highly sensitive detection of a variety of rare cancer-associated antigens (e.g. tumour markers and those of metastatic/residual disease) in complex mixtures and/or antigens contained in small tissue specimens.

### Expression of the cytoplasmic proteins α-catenin and β-catenin in primary and metastatic colorectal cancer: evidence of altered cellular adhesion

*TJ Hugh, GJ Poston and AR Kinsella*

Cellular Oncology Group, Department of Surgery, University of Liverpool, Liverpool L69 3BX, UK

The E-cadherin cell adhesion complex plays a fundamental role in maintaining the epithelial cell phenotype and has been shown to function as a tumour suppressor, in vitro. E-cadherin is a transmembrane glycoprotein which associates with several cytoplasmic molecules, including α-catenin and β-catenin. Binding of E-cadherin to the catenins is essential for normal function of the complex and may serve to link it to the actin cytoskeleton.

Reduced expression of E-cadherin and α-catenin have been shown to correlate with de-differentiation and metastatic potential in several human tumours. However, the role of β-catenin in tumours is less clear as it may be involved in cellular signalling in addition to its role in cell adhesion. Reduced expression of cell adhesion molecules in metastases has also been reported. The aim of this study was to document the expression of α-catenin and β-catenin in a large series of colorectal tumours and their corresponding liver metastases.

Immunohistochemical analysis using monoclonal antibodies was performed on paraffin and frozen sections of 47 specimens of primary adenocarcinomas of the colon and rectum and their corresponding liver metastases.

Only 9/47 (19%) primary tumours and 5/47 (11%) secondary tumours had normal membranous expression of α-catenin. In these cases expression was localised to the apicolateral region of the cell membrane. In 25/47 (53%) primary tumours and 16/47 (34%) secondary tumours cytoplasmic and strong membranous expression of α-catenin were seen together. These sections were scored as abnormal but different from sections with weak or absent expression of α-catenin. The proportion of secondary tumours with weak or absent α-catenin expression was significantly less than in the primary tumours (Wilcoxin matched-pairs signed-ranks test, $P < 0.05$). There was no apparent association between the differentiation status or Duke's stage of the primary tumour and expression of α-catenin.

With regard to β-catenin, 39/47 (83%) primary tumours and all of the liver metastases displayed abnormal expression, often with a characteristically intense staining pattern throughout the cytoplasm as well as along the cell–cell boundaries. Intensity of expression of β-catenin was most marked at 'proliferating fronts' of tumour growth and, in general, these areas of cytoplasmic staining corresponded with reduced membranous staining. There was no association between differentiation status or Duke's stage and expression of β-catenin in the primary tumour. Also, no correlation was found between expression of α-catenin or β-catenin in the primary tumours and overall survival.

Conclusions: Approximately 80% of primary colorectal cancers display abnormal expression of α-catenin and β-catenin and expression of these molecules in liver metastases is even less marked. This suggests that cellular adhesion is not re-established in metastatic tumours. The results of this study are currently being compared to another series of unselected patients with colorectal cancer.

### Regulation of tumour associated macrophage MMP-9 production by a TNF-α loop and a matrix metalloprotease inhibitor

*TM Leber and FR Balkwill*

Imperial Cancer Research Fund London, 44 Lincoln's Inn Fields, London WC2A 3PX

The matrix metalloprotease MMP-9 (gelatinase B) localises to tumour associated macrophages in ovarian cancer, but the signals within the tumour leading to its production are not defined. Co-culture of ovarian cancer cells (PEO-1) and the monocytic cell line (THP-1) led to production of the 92 kDa form of MMP-9. Conditioned medium (CM) derived from PEO-1 also stimulated monocytic cells to produce MMP-9 but not vice versa. There was evidence that TNF-α was involved in tumour stimulated monocyte MMP-9 production: antibody to TNF-α inhibited MMP-9 production in the co-cultures and by CM treated monocytes. In addition,

 *© Cancer Research Campaign 1997*

the synthetic matrix metaloprotease inhibitor (MMPI) BB-2116, which also blocks TNF-α shedding, inhibited MMP-9 release in the co-cultures, and, at the same $IC_{50}$, from CM stimulated monocytic cells. However, two different experiments suggested that the stimulating factor present in CM was not TNF-α. We suggest that ovarian cancer cells can stimulate monocytic cells to make MMP-9 via a so far unidentified factor and that this release is dependent on a monocytic autocrine TNF-α loop on the monocytic cells. Synthetic MMPIs may indirectly inhibit protease production by preventing autocrine or paracrine cytokine release. This may contribute to their actions in models of cancer and inflammatory disease.

### Induction of spheroid formation in breast cancer cell lines by antibody 17E6 requires not only functional E-cadherin but also both inhibition and crosslinking of αv integrins

*John F Marshall, Philip Perry and Ian R Hart*

Richard Dimbleby/ICRF Department of Cancer Research, St Thomas's Hospt., London SE1 7EH, UK

We are investigating the role of αv integrins in the growth and behaviour of breast carcinoma cells. Less than 1 μg/ml of the αv-blocking antibody 17E6 (supplied by S. Goodman, E. Merck) induces formation of multicellular spheroids from monolayers of the breast cell lines ZR75-1, BT474 and T47D, all of which express functional E-cadherin. In contrast, 5 μg/ml of 17E6 has little effect on the breast lines MCF7, MDAMB 231, MDAMB 468, SkBr3 and BT20, all of which lack functional E-cadherin. Pre-treatment of ZR75-1 cells with the E-cadherin blocking antibody DECMA-1 inhibited the spheroid-inducing effects of 17E6. These data show that functional E-cadherin is required for spheroid formation and suggest that there may be 'cross-talk' between E-cadherin and integrins. Monolayers of ZR75-1 cells did not form spheroids in response to whole IgG of the non-blocking anti-αv antibodies 13C2 or P2W7, but did form spheroids in response to bivalent F(ab)2 fragments of 17E6. Monovalent F(ab) fragments of 17E6 were unable to induce spheroid formation unless goat anti-mouse antiserum was also present. As both 17E6 F(ab)2 and 17E6 F(ab) fragments block αv these data suggest that neither blocking αv nor αv-crosslinking alone is sufficient for 17E6-induction of spheroids and that both events are required. Moreover, since integrin-crosslinking is associated with optimal integrin signalling these data indicate that integrin-mediated signalling may have profound effects on the morphogenesis of breast cancer cells.

### Retroviral transfer of the integrin β3 cDNA into β3 negative human melanoma cells

*H Cadiou, JF Marshall, IR Hart*

Richard Dimbleby Department of Cancer Research, Imperial Cancer Research Fund Laboratory, St Thomas's Hospital, London SE1 7EH

The αvβ3 integrin is a cell surface adhesion glycoprotein thought to be involved in the migration and metastasis of malignant melanoma cells. The mechanistic basis of this involvement has not been fully defined. To clarify the role of αvβ3 in the invasive and malignant behaviour of melanoma we have carried out a β3 transfection study. Two αvβ3-negative human occular melanoma cell lines, VUP and OM431 (expressing αvβ1 and αvβ5), were infected with a retrovirus containing the full-length β3 cDNA under the transcriptional control of the strong viral LTR promoter. As controls, retroviruses lacking the gene or containing a mutant, non-functional β3 integrin were employed. Puromycin-resistant colonies were selected and β3-expressing clones were isolated by Dynabead sorting using the anti-β3 monoclonal antibody SZ.21. FACS analysis with the heterodimer-specific antibodies LM609 and 23C6 verified cell surface expression of αvβ3. Immunoprecipitation with the LM609 antibody confirmed *de novo* expression of αvβ3 heterodimers in the infected cells. Unexpectedly the monomer β3 seemed to show a greater affinity for αv than did β1 as indicated by the reduction in or lack of detectable αvβ1 by immunoprecipitation observed in both VUP and OM431 infected cells. These data are consistent with the possibility that αvβ3 expression in the later stages of melanoma development is a consequence of the 'switching on' of β3 expression and the preferential formation of αvβ3 heterodimers.

### Evidence for a tumour suppressor gene on distal chromosome 17q with early effects in ovarian cancer

*I Hickey[1], S McIlroy[1], AN Cranston[1], J McCormick[1], P Harkin[1], RJ Atkinson[2], J Weissenbach[3], EJ Stanbridge[4] and SEH Russell[2]*

[1]School of Biology and Biochemistry, and [2]School of Clinical Medicine, Queen's University, Belfast, N. Ireland, [3]Genethon, Every, France, [4]University of California, Irvine, USA

We have analysed a bank of 75 sporadic epithelial ovarian tumours including 16 benign samples for loss of heterozygosity at 20 loci on chromosome 17. The majority of malignant tumours showed extensive loss involving the whole chromosome or at least the entire long arm. However, a sub-set of tumours showed distinct regions of interstitial loss. Although some examples of partial deletions were found on the short arm, most involved the long arm. A common region of deletion spanning less than 1 cM lying between D17S939 and D17S937 was observed in eight tumours. These included examples of all the major histopathological classes of ovarian tumours, serious, endometrioid and mucinous.

Significantly this group included two benign tumours and one borderline tumour. This indicates that loss from this region may represent an early event in the development of epithelial ovarian tumours, which is often masked by subsequent losses from other regions of this chromosome. This common region of deletion is clearly distal to BRCA1, and FISH analysis using YACs identified as carrying microsatellites mapping to the region indicates a location close to the telomere.

We have also attempted to find functional evidence for the presence of a tumour suppressor gene by using microcell fusion to introduce a normal copy of chromosome 17 into ovarian cell lines. Two cell lines, A2780 and OAW42 were used. In both cases clones of hybrid cells were formed which ceased growth within seven weeks after fusion. We have excluded p53 as being the gene responsible for this growth suppression, but we have not yet mapped the gene responsible to a specific region of the chromosome.

In conclusion we have produced evidence for a specific region of loss of heterozygosity in ovarian tumours which can be detected at early stages, and the presence of a gene on the same chromosome which is responsible for regulation of immortalisation of ovarian epithelium.

© Cancer Research Campaign 1997

### MAGE gene expression in benign and malignant ovarian tissue

*AM Gillespie[1], S Rodgers[1], AP Wilson[2] and RC Rees[3]*

[1]YCRC Inst. Cancer Studies and Dept. Clinical Oncology, University of Sheffield, Sheffield S10 2RX, [2]Oncology Research Lab., Derby City Gen. Hospital, Derby DE22 3NE, [3]Dept. Life Sciences, Nottingham Trent University, Nottingham N11 8NF

In the UK ovarian cancer is the most common gynaecological malignancy. Due to the nature of the condition and the limitations of current therapy, prognosis is poor with an overall 5 year survival of only 30%. New treatment modalities may, in future, contribute to an improved outlook for women diagnosed with this disease.

One such therapy may be Antigen Specific Immunotherapy with MAGE peptide products. It is known that MAGE 1–4, 6 & 12 genes are expressed in some human cancers,[1] but not normal tissues with the exception of the testes and placenta. MAGE 1 & 3 are targets for specific immunotherapy as they encode peptide antigens – 'tumour antigens' – which are presented in association with HLA class 1 molecules and are recognised by cytotoxic T lymphocytes.

To determine if ovarian cancer patients would be suitable for MAGE-peptide vaccine based immunotherapy, the frequency of MAGE 1–4 genes expression in ovarian tumours was assessed using Reverse Transcription Polymerase Chain Reaction (RT-PCR) and product verification with digoxigenin-labelled oligonucleotide probes specific for each MAGE gene.

In this preliminary study 12/20 ovarian cancer specimens studied expressed MAGE 1, including 8/9 serous adenocarcinomas. In addition 13/19 benign pathological ovarian lesions studied also expressed MAGE 1, including simple cysts, cystadenomas and adenofibromas. No MAGE expression was detected in 5 normal ovarian specimens.

If the frequency of MAGE 1 expression in serious cystadenocarcinomas is confirmed in this ongoing study, patients with this condition will be candidates for MAGE-peptide immunotherapy.

It has previously been asserted that MAGE gene expression is a relatively late event in the neoplastic transformation of cells.[2] Our initial findings suggest that MAGE 1 is expressed in non-malignant ovarian tissue. Further exploration of MAGE expression in benign and malignant ovarian tumours may shed new light on the poorly understood natural history of ovarian carcinoma.

#### REFERENCES

1. P Van Der Bruggen et al (1991). A gene encoding an antigen recognised by cytotoxic T lymphocytes on a human melanoma. Science 54: 1643
2. F Brasseur et al (1995). Expression of MAGE genes in primary and metastatic cutaneous melanoma. Int J Cancer 67: 54

### A quantitative assessment of the leukocyte infiltrate in ovarian cancer and its relationship to the expression of c-c chemokines

*RPM Negus[1], GWH Stamp[2], J Hadley[3] and FR Balkwill[1]*

[1]Imperial Cancer Research Fund, London WC2A 3PX, [2]Hammersmith Hospital, London W12 0HS, [3]ICRF, Oxford OX3 7LF

We have defined the host leukocyte infiltrate in epithelial ovarian tumours and related this to the expression of c-c chemokines. Immunohistochemical analysis of 20 paraffin embedded biopsies showed that the infiltrate was primarily composed of CD68+ macrophages and CD8+/CD45RO+ T-cells (median values 3700 cells/mm³ and 2200 cells/mm³ respectively). Natural killer cell, B-cells, and mast cells occurred in lower numbers (median values 0–200 cells/mm³). Eosinophils were rarely seen and neutrophils were mainly confined to blood vessels. More infiltrating cells were found in stromal than in tumour areas. Only macrophages occurred in significant numbers in areas of necrosis ($P < 0.0005$). Using in situ hybridisation to mRNA we examined expression of the chemokines MCP-1, MIP-1α, MIP-1β and RANTES. MCP-1 and MIP-1α were expressed by significantly more cells than MIP-1β and RANTES ($P < 0.005$). In tumour epithelial areas, the predominant chemokine was MCP-1. MCP-1 and MIP-1α were the predominant stromal chemokines. A significant correlation was found between the total number of CD68+ macrophages or CD8+ T-cells and the number of cells expressing MCP-1 ($r_s = 0.50$, $P = 0.026$ and $r_s = 0.63$, $P = 0.003$ respectively) and also between the CD8+ population and RANTES expressing cells ($r_s = 0.6$, $P = 0.003$). We suggest that MCP-1 is largely responsible for the leukocyte infiltrate in ovarian carcinomas, but the expression of other chemokines may determine its exact nature. We have begun to examine the factors that may be responsible for the expression of MCP-1 in these tumours. Using RT-PCR and Northern analysis, we have found that TNF-α stimulated tumour cell expression of MCP-1. Maximal expression was at about 3 hours, with levels falling over 24 hours. Since TNF-α is expressed by both tumour cells and tumour associated macrophages and can stimulate MCP-1 expression in a variety of cell types, this cytokine provides one possible link between the infiltrating cell population and the pattern of expression of chemokines observed within ovarian carcinomas.

### Mismatch repair deficiency and cisplatin resistance in ovarian cancer

*A Anthoney[1], A McIlwrath[1], A van der Zee[2], G Hirst[1], R Brown[1]*

[1]CRC Dept. of Medical Oncology, CRC Beatson Laboratories, Garscube Estate, Glasgow G61 1BD. [2]Dept. Gynecologic. Oncology, University Hospital, Groningen, The Netherlands

We have previously shown that nine of ten independent cisplatin resistant derivatives of an ovarian carcinoma cell line possess a microsatellite instability phenotype.[1] The lines also display cross-resistance to the methylating agent methylnitrosourea (MNU) after inhibition of alkyltransferase activity with O⁶benzylguanine. Two of these lines have subsequently been shown to be defective in strand specific mismatch repair of single base and dinucleotide mismatches using an in vitro mismatch repair assay.[2] Complementation of cell extracts with purified mismatch repair proteins identified hMutLα as the defective component of mismatch repair in these lines.

To further define the defect in mismatch repair in the set of ten cisplatin resistant cell lines we have determined the levels of the four major mismatch repair proteins. Using a Western immunoblot assay no significant variation in the levels of hMSH2 or GTBP (hMSH6) were observed amongst the cell lines or when compared to the parental line. However, no hMLH1 or hPMS2 protein was detectable in nine of the resistant cell lines. Immunohistochemistry confirmed the absence of hMLH1 and hPMS2 protein expression in the resistant derivatives compared to parental cells. RT-PCR

© Cancer Research Campaign 1997

was used to assess mRNA expression from the mismatch repair genes. By this method levels of hMSH2 and hPMS2 mRNA were similar in all lines. One of the cisplatin resistant lines showed similar levels of hMLH1 mRNA to parental cells whilst in another the levels, although detectable, were greatly reduced. No hMLH1 mRNA was detectable in any of the other 8 cisplatin resistant lines. Southern analysis of the hMLH1 gene between parental and resistant lines revealed no gross rearrangement to explain the lack of hMLH1 expression. In conclusion, lack of hMLH1 gene expression is the basis for the defective mismatch repair in a series of cisplatin resistant ovarian carcinoma cell lines.

We have, in addition, observed loss of hMLH1 expression in 10% (4/41) of untreated ovarian tumours and 30% (4/12) of residual tumour removed at second look surgery after platinum based chemotherapy. This more frequent loss of hMLH1 post chemotherapy is statistically significant ($P < 0.05$). No difference was observed for hPMS2, hMSH2 or GTBP.

**REFERENCES**

1. Anthoney DA et al Cancer Research 56, 1374–81, 1996
2. Drummond JD et al Journal Biological Chemistry. 271, 19645–48, 1996

*© Cancer Research Campaign 1997*